# KLF6 Acetylation Promotes Sublytic C5b-9-Induced Production of MCP-1 and RANTES in Experimental Mesangial Proliferative Glomerulonephritis

**DOI:** 10.7150/ijbs.46573

**Published:** 2020-06-20

**Authors:** Tianyi Yu, Yajuan Gong, Yu Liu, Lu Xia, Chenhui Zhao, Longfei Liu, Mengxiao Xie, Zhijiao Wu, Dan Zhao, Wen Qiu, Yingwei Wang, Jing Zhang, Mingde Ji

**Affiliations:** 1 Department of Immunology, Key Laboratory of Immunological Environment and Disease, Nanjing Medical University, Nanjing, Jiangsu 211166, China.; 2 Department of Oncology, the First Affiliated Hospital of Nanjing Medical University, Nanjing, Jiangsu 210029, China.; 3 Key Laboratory of Antibody Technology of Ministry of Health, Nanjing Medical University, Nanjing, Jiangsu 211166, China.; 4 Department of Laboratory Medicine, Jiangsu Province Hospital of Chinese Medicine, Affiliated Hospital of Nanjing University of Chinese Medicine, Nanjing, Jiangsu 210029, China.

**Keywords:** KLF6, acetylation, sublytic C5b-9, MCP-1, RANTES, Thy-1 nephritis

## Abstract

Rat Thy-1 nephritis (Thy-1N) is an experimental mesangial proliferative glomerulonephritis (MsPGN) for studying human MsPGN. Although sublytic C5b-9 complex formation on glomerular mesangial cells (GMCs) and renal MCP-1 and RANTES production in rats with Thy-1N have been proved, the role and mechanism of MCP-1 or RANTES synthesis in GMCs induced by sublytic C5b-9 are poorly elucidated. In this study, we first found the expression of transcription factor (KLF6), co-activator (KAT7) and chemokines (MCP-1 and RANTES) was all up-regulated both in renal tissue of Thy-1N rats (*in vivo*) and in sublytic C5b-9-induced GMCs (*in vitro*). Further *in vitro* experiments revealed that KLF6 bound to MCP-1 promoter (-297 to -123 nt) and RANTES promoter (-343 to -191 nt), leading to MCP-1 and RANTES gene transcription. Meanwhile, KAT7 also bound to the same region of MCP-1 and RANTES promoter in a KLF6-dependent manner, and KLF6 was acetylated by KAT7 at lysine residue 100, which finally promoted MCP-1 and RANTES expression. Moreover, our* in vivo* experiments discovered that knockdown of renal KAT7 or KLF6 gene obviously reduced MCP-1 and RANTES production, GMCs proliferation, ECM accumulation, and proteinuria secretion in Thy-1N rats. Collectively, our study indicates that sublytic C5b-9-induced MCP-1 and RANTES synthesis is associated with KAT7-mediated KLF6 acetylation and elevated KLF6 transcriptional activity, which might provide a new insight into the pathogenesis of rat Thy-1N and human MsPGN.

## Introduction

Human mesangial proliferative glomerulonephritis (MsPGN), one of the most common forms of glomerulonephritis worldwide, is more frequently found in IgA nephropathy 1. Histologically, MsPGN is characterized by renal inflammation, glomerular mesangial cells (GMCs) proliferation, and extracellular matrix (ECM) expansion 2-4. Rat Thy-1 nephritis (Thy-1N) is an animal model for studying MsPGN, which can be induced by injection of antibody to Thy-1 antigen expressed on the membrane of rat GMCs, and then activating the complement system and leading to C5b-9 complex formation 5. Many studies have confirmed that the pathologic changes, such as inflammatory cytokines production and GMCs damage, are C5b-9-dependent, especially sublytic C5b-9-dependent in rat Thy-1N 6-8. GMCs exposed to sublytic C5b-9 *in vitro* can significantly elevate the synthesis of IL-23, IL-36a 7 and IL-6 8. Because several documents have pointed out that some inflammatory chemokines i.e. MCP-1 and RANTES are increased in glomerulus of MsPGN patients and Thy-1N rats 9-14, thus whether sublytic C5b-9-attacked rat GMCs can produce MCP-1 or RANTES, and its molecular mechanism relevant for MCP-1 and RANTES gene transcription in Thy-1N need to be further determined.

In recent years, numerous experiments have focused on the role of some transcription factors in the regulation of MCP-1 or RANTES gene expression 9, 15. Kruppel-like factor 6 (KLF6), as a transcription factor belonging to the KLF family, is a ubiquitously expressed zinc finger protein and possesses a NH2 terminus activation domain and a COOH terminus DNA-binding domain that binds to ''GC box'' or ''CACCC elements” in responsive promoters 16-18. Reportedly, KLF6 participates in TGFβ1-induced epithelial-mesenchymal transition in proximal tubule cells, which promotes renal injury and fibrosis 19. Moreover, KLF6 regulates the expression of macrophage inflammatory protein-3α, which attracts macrophages infiltrate into the tubulointerstitium causing kidney inflammation in diabetic rat 20. Our previous microarray analysis has revealed that KLF6 is greatly up-regulated in sublytic C5b-9-treated GMCs 21 and computer-assisted analysis also shows the putative KLF6 binding sites on MCP-1 and RANTES promoter, but whether KLF6 is involved in the modulation of MCP-1 and RANTES gene transcription in GMCs attacked by sublytic C5b-9 in Thy-1N rats remains unclear.

Generally, transcription factor regulate target gene transcription by recruiting chromatin modifier, co-factor, and transcription machinery to gene promoter. It has demonstrated that a number of KLFs bind to co-factors that possess histone acetyltransferase (HAT) activity, such as cAMP response element binding-binding protein (CBP), p300, and p300/CBP-associated factor (P/CAF) 7, 22, 23. As a co-factor, lysine acetyltransferase 7 (KAT7, also known as HBO1 or MYST2) is also a member of the HAT family 24, and KAT7 is involved in gene transcription via acetylation of histone H3/H4 and non-histone proteins 25-27. Besides, KAT7, as a regulator of inflammatory cytokine gene transcription, not only affects IL-1β, IL-6, and IL-10 synthesis in THP-1 monocytes upon lipopolysaccharide exposure, but also induces IL-6 expression in synovial fibroblasts via epigenetic mechanism 28, 29. However, the effect of KAT7 on regulating KLF6 function in MCP-1 and RANTES production in GMCs attacked by sublytic C5b-9 of Thy-1N rats has not been explored.

In order to solve these problems described previously, in the present study, we first detected and compared the expression levels and phases of KLF6, KAT7, MCP-1 and RANTES both in renal tissue of Thy-1N rats (*in vivo*) and in GMCs stimulated with sublytic C5b-9 (*in vitro*). Next, we determined *in vitro* the role and mechanism of KLF6 and KAT7 in MCP-1 and RANTES gene transcription. Additionally, we investigated *in vivo* the effect of renal KAT7 or KLF6 gene knockdown on the production of MCP-1 and RANTES, glomerular pathological change, and proteinuria secretion of Thy-1N rats.

## Materials and Methods

### Serum, complement and antibodies

Normal human serum (NHS) pooled from 30 healthy adult donors were used as a source of complement. Heat-inactivated serum (HIS) was obtained by incubating NHS at 56°C for 30 min. Human C6-deficient serum (C6DS) was from Sigma-Aldrich. Recombinant human C6 was from Sino Biological. Rabbit polyclonal Thy-1 antibody (Ab, 1:640) and normal rabbit serum were prepared in our laboratory according to previous document 30. Ab against KLF6 (sc-365633) was from Santa Cruz Biotechnology. Ab against KAT7 (ab190908) was from Abcam. Ab against acetylated-lysine (Ac-k; #9681) was provided Cell Signaling Technology.

### Plasmids construction and lentivirus packaging

The pIRES2-KLF6 and pIRES2-KAT7 were constructed by inserting complete open reading frames of rat KLF6 (NM_031642.4) and KAT7 (NM_181081.2) gene into pIRES2-EGFP vector, respectively. FLAG-tag was added to the 5′ of KLF6. Primer sequences are listed in Table S1. KLF6 mutants (K100R, K213R, K100/213R, and K100Q) were custom produced by Genscript.

The shKLF6 and shKAT7 were constructed using pGCsi-U6/Neo/GFP vector. The shRNA targeted sequences are as follows: shKLF6, CTTAGTCAATTCAGGAAAT; shKAT7, GGAGAAGTTAAGGCTTCAAGG. Additionally, lentivirus (LV) products for transduction of shKLF6 (LV-shKLF6) and shKAT7 (LV-shKAT7) were custom produced by GenePharma.

The pGL3-MCP-1-full length (FL) was constructed by inserting rat MCP-1 (Gene ID: 24770) promoter fragment -1670 to -30 nt into pGL3-basic vector. The promoter deletion fragments were cloned into the same vector: truncation 1 for -1508 to -30 nt, truncation 2 for -872 to -30 nt, truncation 3 for -297 to -30 nt and truncation 4 for -123 to -30 nt. The pGL3-RANTES-FL was constructed by inserting rat RANTES (Gene ID: 81780) promoter fragment -1744 to -14 nt into pGL3-basic vector. Four promoter deletion fragment plasmids were also constructed: 1 for -1464 to -14 nt, 2 for -837 to -14 nt, 3 for -343 to -14 nt and 4 for -191 to -14 nt. Primer sequences are shown in Table S1.

### Animal experiments

Male SD rats (170-200 g) were from B&K Universal Ltd (Shanghai, China). Rats were maintained in animal facilities under pathogen-free conditions, and the animal study was approved by the Animal Ethical and Welfare Committee of Nanjing Medical University. In some experiments, SD rats were divided into 2 groups (n=6 in each time point): Thy-1N group rats were injected intravenously with Thy-1 Ab (1 ml/100 g) by an intravenous injection; NS group rats were injected intravenously with normal rabbit serum (1 ml/100 g).

In other experiments, LV-shRNA was infused into rat kidney via renal artery perfusion, immediately followed by renal vein occlusion for 10 min as previously described 31. GFP was observed to define the efficiency of transferring LV-shRNA into kidneys by an IVIS *in vivo* imaging system (Caliper Life Sciences). Then 96 h later, Thy-1N was established as mentioned above. Accordingly, SD rats were divided into 5 groups (n=6 in each group): NS, Thy-1N, LV-shCTR + Thy-1N, LV-shKLF6 + Thy-1N, and LV-shKAT7 + Thy-1N. Renal cortex samples were obtained after the rats were euthanized at the indicated time points.

### Sublytic C5b-9 determination and GMCs treatment

Rat GMCs strain (HBZY-1) was obtained from the China Centre for Type Culture Collection. GMCs were cultured in MEM (Gibco) containing 10% FBS (Wisent). Lactate dehydrogenase (LDH) was detected in the supernatants of cultured cells, and < 5% LDH release from cells was regarded as sublytic 32, 33. According to this criterion, 5% Thy-1 Ab plus 4% NHS were used to assemble sublytic C5b-9 on GMCs membrane as previously described 34. GMCs were also treated into other groups: Thy-1 Ab group represented GMCs incubated only with 5% Thy-1 Ab; Thy-1 Ab + HIS group represented 5% Thy-1 Ab-sensitized GMCs incubated with 4% HIS; Thy-1 Ab + C6DS group represented 5% Thy-1 Ab-sensitized GMCs incubated with 4% C6DS; Thy-1 Ab + C6DS + C6 group represented 5% Thy-1 Ab-sensitized GMCs incubated with 4% C6DS and then reconstituted with 2mg/l C6.

### Cell transfection and infection

GMCs were transiently transfected with corresponding plasmids by using the Neon Transfection System (Invitrogen) as previously mentioned 8. Besides, 0.5 × 10^4^ GMCs were infected with LV-shRNA at the titer of 1 × 10^6^ or 1 × 10^7^ TU/ml for 48 h as previous document 7.

### Quantitative PCR (qPCR) and reverse transcription PCR (RT-PCR) assays

Total RNA was extracted from renal cortex or GMCs using TRIzol reagent (Invitrogen) and reverse-transcribed into cDNA using HiScript 1st Strand cDNA Synthesis Kit (Vazyme), according to the manufacturer's protocol. The qPCR assay was performed using AceQ qPCR SYBR Green Master Mix (Vazyme), and RT-PCR assay was performed using 2 × Taq Master Mix (Vazyme). Primer sequences are shown in Table S2. Data were normalized to β-actin or GAPDH expression.

### Enzyme linked immune sorbent assay (ELISA)

Homogenization and subsequent procedures were performed at 4°C. In brief, renal tissues (100 mg) were homogenized in 1 ml pre-cooling PBS containing 0.25 mol/l sucrose, 1 mmol/l EDTA, and 0.25 mmol/l PMSF and then centrifuged at 120,000 ×g for 30 min. MCP-1 and RANTES levels in the supernatants from renal cortex or GMCs were measured using ELISA kits (Shanghai Enzyme-linked Biotechnology Co.) according to the manufacturer's recommendations.

### Immunoblotting (IB) analysis

Renal cortex or GMCs lysates were prepared in lysis buffer (Cell Signaling Technology) and additional Protease Inhibitor Cocktail (Abcam). Equal amounts of proteins were counted after concentrations measured with the BCA Protein Assay Kit (Pierce). Samples were separated by SDS-PAGE and transferred to PVDF membranes (Millipore), blocked with 5% nonfat dried milk and incubated overnight at 4°C with specific primary Abs. Corresponding secondary Abs were subsequently applied, and the signals were detected using the Amersham imager 600 (GE).

### Immunoprecipitation (IP) analysis

Renal cortex or GMCs lysates were incubated overnight at 4°C with specific primary Abs. The mixture was captured with protein A/G plus-agarose beads (Santa Cruz Biotechnology). After being washed extensively, samples were analyzed by IB as described above.

### Luciferase reporter assay

GMCs were transiently transfected with the indicated combinations of plasmids and then stimulated with or without sublytic C5b-9. Cell extracts were prepared, and luciferase activity was measured using the Dual-Luciferase Reporter Assay System (Promega). The firefly luciferase activity was normalized to renilla luciferase.

### Chromatin immunoprecipitation (ChIP) and Re-ChIP assays

GMCs were cross-linked with 1% formaldehyde and subjected to ChIP assay following the instructions of the Chromatin Immunoprecipitation Assay Kit (Millipore). For Re-ChIP assay, the primary ChIP material was released from the agaroses and subjected to second round of IP using corresponding Ab. Purified DNA was analyzed by RT-PCR and qPCR, and the primers are shown in Table S2. Data were normalized to input DNA.

### Mass spectrometry analysis

GMCs were transfected with pIRES2-KLF6-FLAG vector and then stimulated with or without sublytic C5b-9. Cell lysates were bound to anti-FLAG immunoaffinity resin (Sigma-Aldrich), and then the bounded protein complexes were competitively eluted using synthetic flag peptide. The purified KLF6-FLAG fusion protein was analyzed by mass spectrometer Ultraflex II (Bruker) at Center of Hygienic Analysis & Detection of Nanjing Medical University.

### Histological examination

Renal cortex paraffin sections (4 μm) on day 7 after Thy-1N establishment were stained with H&E. The number of glomerular cells was quantified from the counts of positive-stained nuclei, which was performed in a double-blinded manner by two independent observers under light microscope. Ultrastructural changes of glomerular lesions were examined by transmission electron microscopy.

### Urine protein detection

The urinary protein (mg/24 h) of Thy-1N rats on day 7 was measured by the Total protein UC FS (DiaSys Diagnostic Systems).

### Statistical analysis

All experiments were performed in triplicate and data are presented as mean ± SD. Statistical significance, defined as P < 0.05, was evaluated using one-way ANOVA or Student's t-test.

## Results

### Production of MCP-1 and RANTES is increased both in renal tissue of Thy-1N rats and in GMCs stimulated with sublytic C5b-9

**Figure 1:**
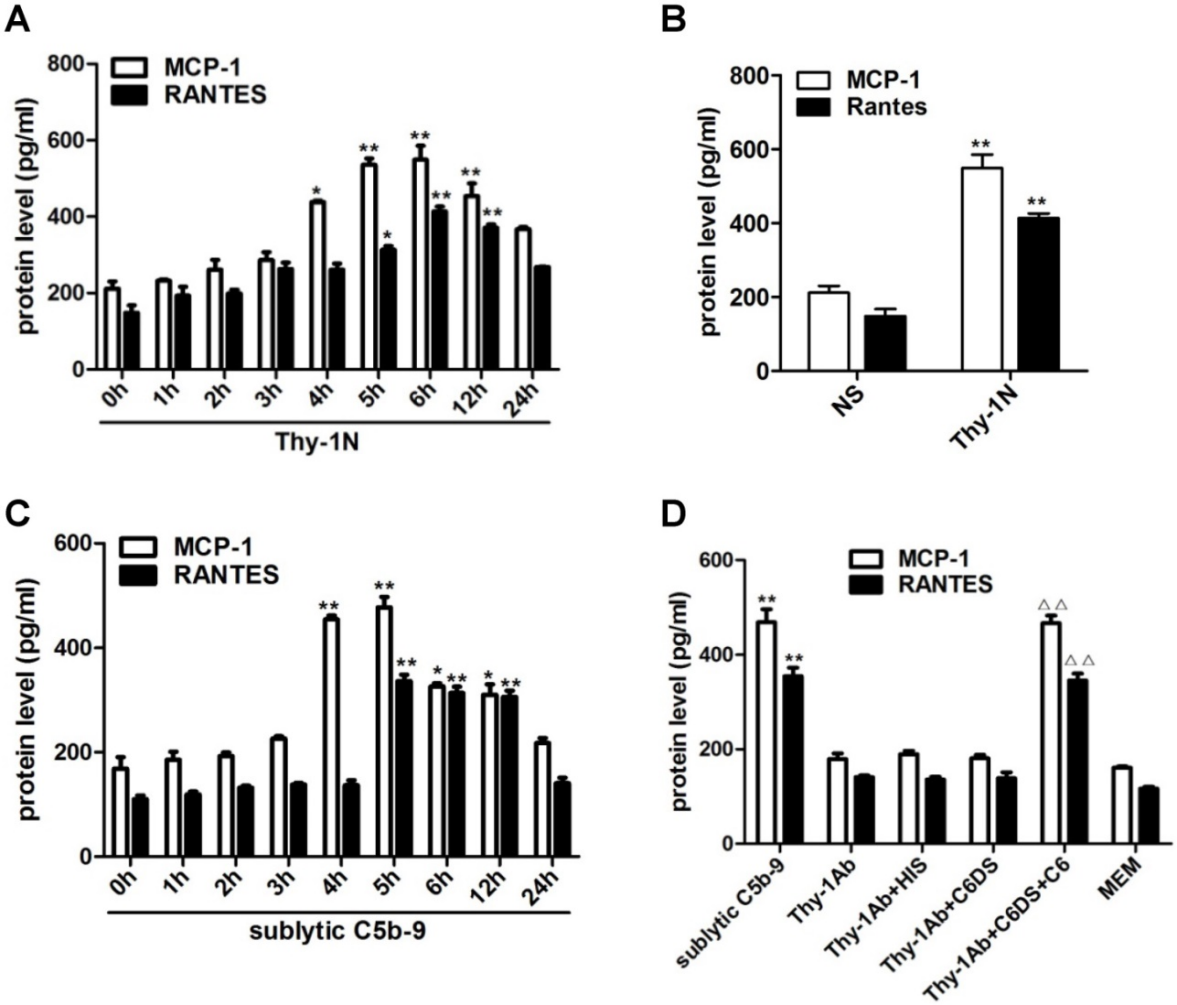
Expression of MCP-1 and RANTES both in renal tissue of Thy-1N rats and in GMCs upon sublytic C5b-9 stimulation. **(A)** ELISA of MCP-1 and RANTES in renal cortex supernatants of SD rats injected intravenously with Thy-1 Ab for the indicated times (*P < 0.05, **P < 0.01 vs. 0 h). **(B)** ELISA of MCP-1 and RANTES in renal cortex supernatants of SD rats injected intravenously with Thy-1 Ab or normal rabbit serum (NS) for 6 h (**P < 0.01 vs. NS). **(C)** ELISA of MCP-1 and RANTES in supernatants of GMCs stimulated with sublytic C5b-9 for the indicated times (*P < 0.05, **P < 0.01 vs. 0 h). **(D)** ELISA of MCP-1 and RANTES in supernatants of GMCs stimulated with sublytic C5b-9, Thy-1 Ab, Thy-1 Ab + HIS, Thy-1 Ab + C6DS, Thy-1 Ab + C6DS + C6, or MEM for 5 h (**P < 0.01 vs. Thy-1 Ab, Thy-1 Ab + HIS, Thy-1 Ab + C6DS, and MEM; ^△△^P < 0.01 vs. Thy-1 Ab + C6DS). Data are shown as mean ± SD from three independent experiments.

Since inflammatory cellular processes in rat Thy-1N are sublytic C5b-9-dependent, and macrophage influx is present in the injured kidney of Thy-1N rats 6, we focused on the relationship between sublytic C5b-9 and the expression of MCP-1 and RANTES. Our data showed that in renal tissue of Thy-1N rats (*in vivo*), the levels of MCP-1 and RANTES secretion were elevated in a time-dependent manner, peaking at 6 h (Figure 1A, 1B). Besides, in GMCs stimulated with sublytic C5b-9 (*in vitro*), the levels of MCP-1 and RANTES were also up-regulated in a time-dependent manner, with maximum secretion occurring at 5 h (Figure 1C). Subsequently, to make sure that MCP-1 and RANTES production is actually due to assembly of sublytic C5b-9 complex, GMCs were treated with sublytic C5b-9 (Thy-1 Ab + NHS), Thy-1 Ab, Thy-1 Ab + HIS, Thy-1Ab + C6DS, and MEM for 5h, respectively. The result exhibited that the expression of MCP-1 and RANTES was markedly increased in sublytic C5b-9 group (Figure 1D). Moreover, adding C6 back to C6DS i.e. Thy-1 Ab + C6DS + C6 treatment could recover the ability to induce MCP-1 and RANTES synthesis (Figure 1D), confirming that MCP-1 and RANTES production is indeed triggered by sublytic C5b-9.

### Expression of KLF6 and KAT7 is up-regulated both in renal tissue of Thy-1N rats and in GMCs attacked by sublytic C5b-9

In order to explore potential mechanisms of MCP-1 and RANTES gene expression, we chose to investigate trans-acting factors such as transcription factors and co-factors. Given that KLF6 overexpression augmented the lipopolysaccharide-induced MCP-1 expression 35, and our previous microarray analysis indicated KLF6 up-regulation in primary GMCs stimulated with sublytic C5b-9 16, we examined KLF6 expression both *in vivo and in vitro*. Meanwhile, we also detected mRNA level of twelve HAT members, which are a specific class of co-factors. Data showed that KAT7 expression was the highest one both *in vivo and in vitro* Figure S1A, S1B).

**Figure 2:**
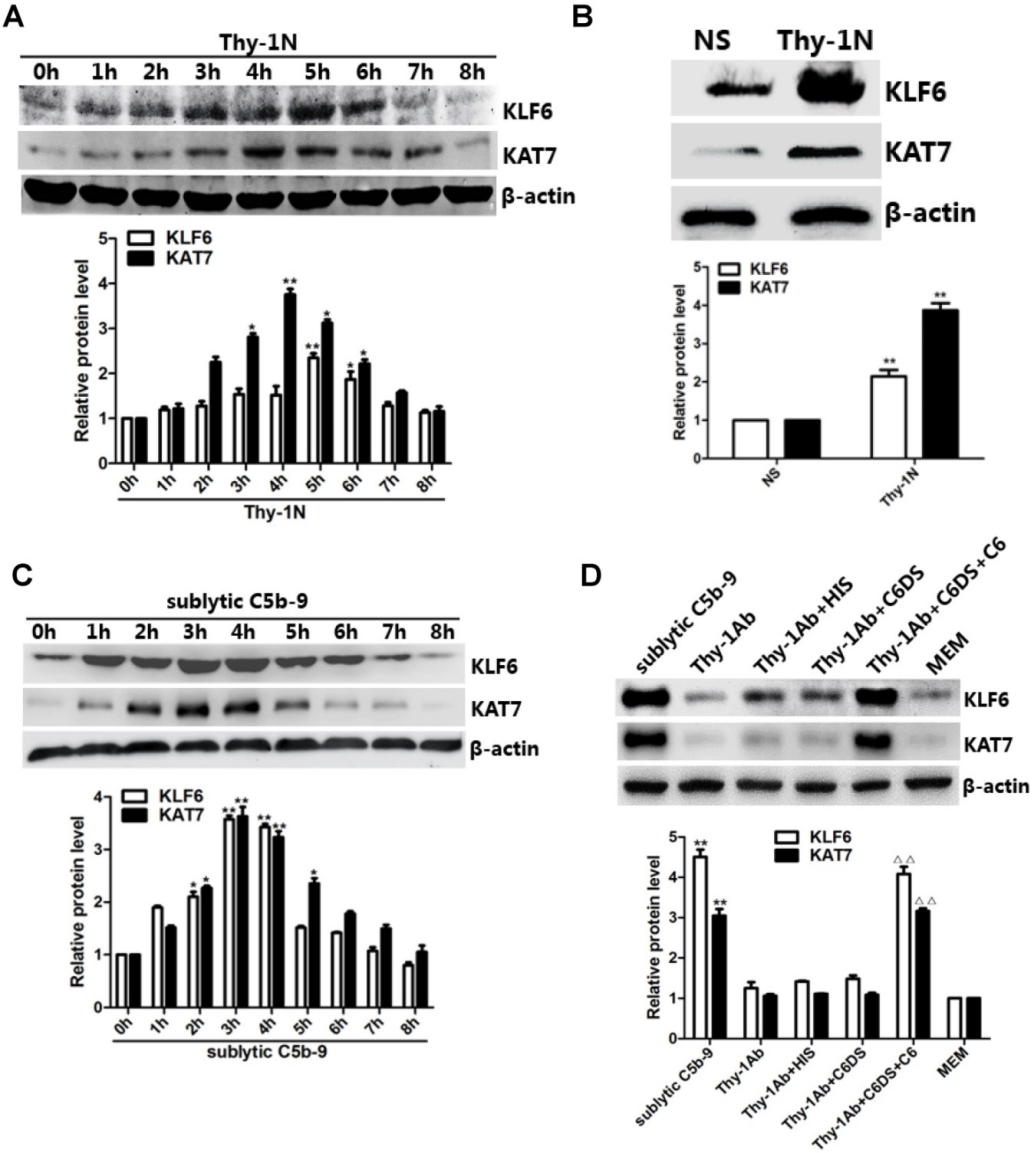
Expression of KLF6 and KAT7 both in renal tissue of Thy-1N rats and in GMCs upon sublytic C5b-9 stimulation. **(A)** IB analysis of KLF6, KAT7, and β-actin in renal cortex of SD rats injected intravenously with Thy-1 Ab for the indicated times (*P < 0.05, **P < 0.01 vs. 0 h). **(B)** IB analysis of KLF6, KAT7, and β-actin in renal cortex of SD rats injected intravenously with Thy-1 Ab or normal rabbit serum (NS) for 4 or 5 h (**P < 0.01 vs. NS). **(C)** IB analysis of KLF6, KAT7, and β-actin in GMCs stimulated with sublytic C5b-9 for the indicated times (*P < 0.05, **P < 0.01 vs. 0 h). **(D)** IB analysis of KLF6, KAT7, and β-actin in GMCs stimulated with sublytic C5b-9, Thy-1 Ab, Thy-1 Ab + HIS, Thy-1 Ab + C6DS, Thy-1 Ab + C6DS + C6, or MEM for 3 h (**P < 0.01 vs. Thy-1 Ab, Thy-1 Ab + HIS, Thy-1 Ab + C6DS, and MEM; ^ΔΔ^P < 0.01 vs. Thy-1 Ab + C6DS). Data are representative of three independent experiments with similar results or are shown as mean ± SD from three independent experiments.

*In vivo* results showed that the mRNA and protein levels of KLF6 and KAT7 were augmented in a time-dependent manner with a peak level at 3-5 h (Figure 2A, 2B, and Figure S1C). In addition, *in vitro* results showed that both mRNA and protein abundance of KLF6 and KAT7 were advanced to a peak at 3 h and gradually declined in a time-dependent manner (Figure 2C, Figure S1D). Moreover, KLF6 and KAT7 expression in response to sublytic C5b-9 or Thy-1 Ab + C6DS + C6 for 3 h was significantly higher than those exposed to other treatments (Figure 2D, Figure S1E). Because KLF6 and KAT7 were expressed at earlier phase than MCP-1 and RANTES did, these data suggest that the production of MCP-1 and RANTES may correlate with increase of KLF6 and KAT7 expression induced by sublytic C5b-9.

### KLF6 and KAT7 are necessary for sublytic C5b-9-triggered MCP-1 and RANTES gene expression

To determine whether KLF6 and KAT7 are necessary and sufficient to induce MCP-1 and RANTES production, overexpression and knockdown approaches were used to investigate gene functions *in vitro*. As shown in Figure S2A-E, shRNA vectors efficiently down-regulated endogenous KLF6 and KAT7 protein, and overexpression plasmids successfully up-regulated exogenous KLF6 and KAT7 protein in rat GMCs.

**Figure 3:**
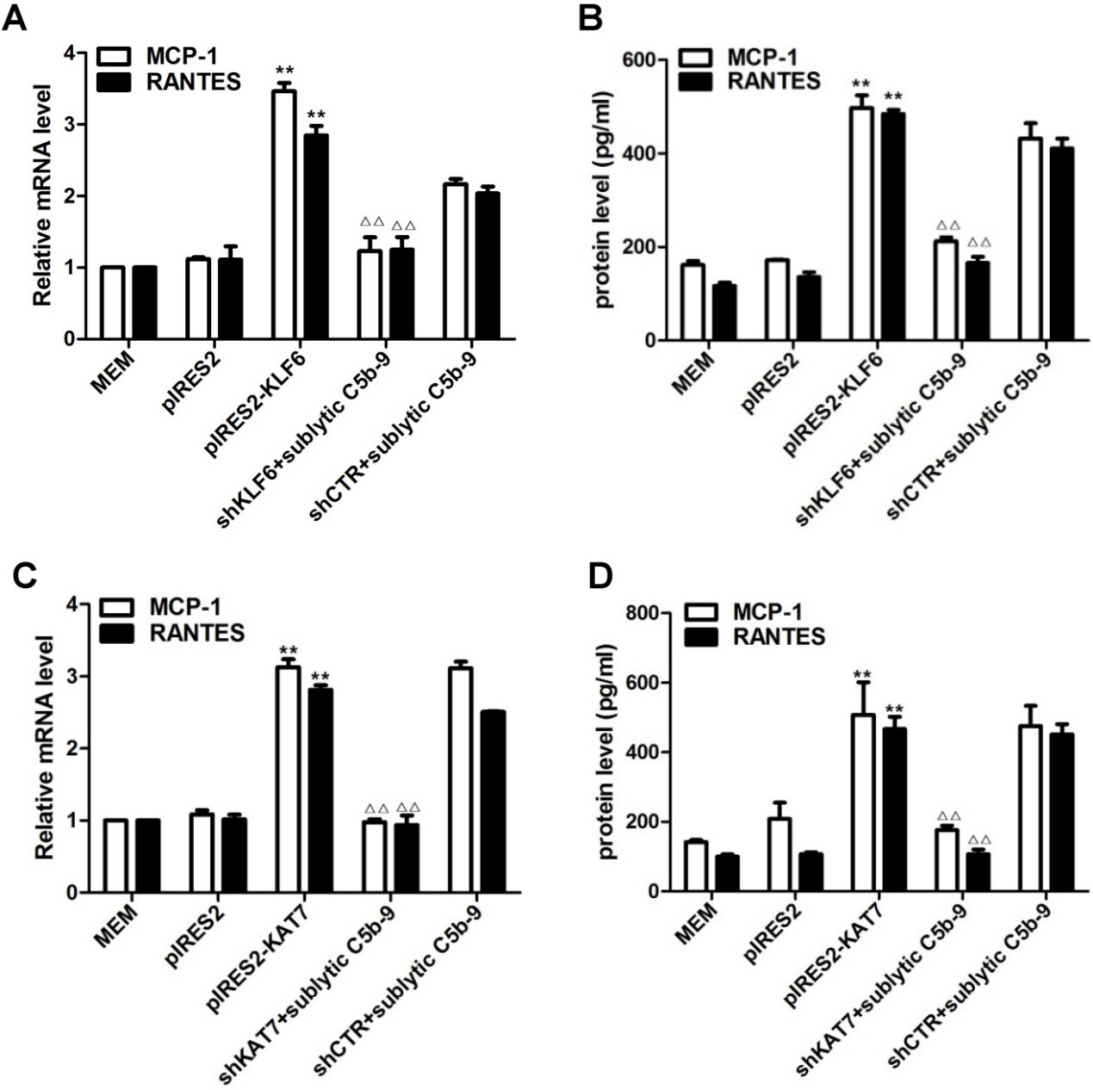
Role of KLF6 and KAT7 expression in sublytic C5b-9-induced MCP-1 and RANTES expression. GMCs were transfected with control vector (pIRES2), vector encoding KLF6 (pIRES2-KLF6) or KAT7 (pIRES2-KAT7), control shRNA (shCTR), or specific shRNA targeting KLF6 (shKLF6) or KAT7 (shKAT7) for 48 h and then incubated with or without sublytic C5b-9 for 5 h. **(A and C)** qPCR analysis of MCP-1 **(A)** and RANTES** (C)** mRNA in GMCs. **(B and D)** ELISA of MCP-1 **(B)** and RANTES **(D)** in supernatants of GMCs. **P < 0.01 vs. pIRES2; ^△△^P < 0.01 vs. shCTR + sublytic C5b-9. Data are shown as mean ± SD from three independent experiments.

Forced KLF6 expression markedly increased mRNA abundance and protein concentration of MCP-1 and RANTES, whereas interference with KLF6 expression notably decreased MCP-1 and RANTES production (Figure 3A, 3B). The effect of KAT7 on MCP-1 and RANTES synthesis was similar to KLF6 (Figure 3C, 3D). Collectively, these findings implicate that KLF6 and KAT7 can somehow be involved in the regulation of MCP-1 and RANTES gene expression.

### KLF6 activates gene transcription of MCP-1 and RANTES together with KAT7

**Figure 4:**
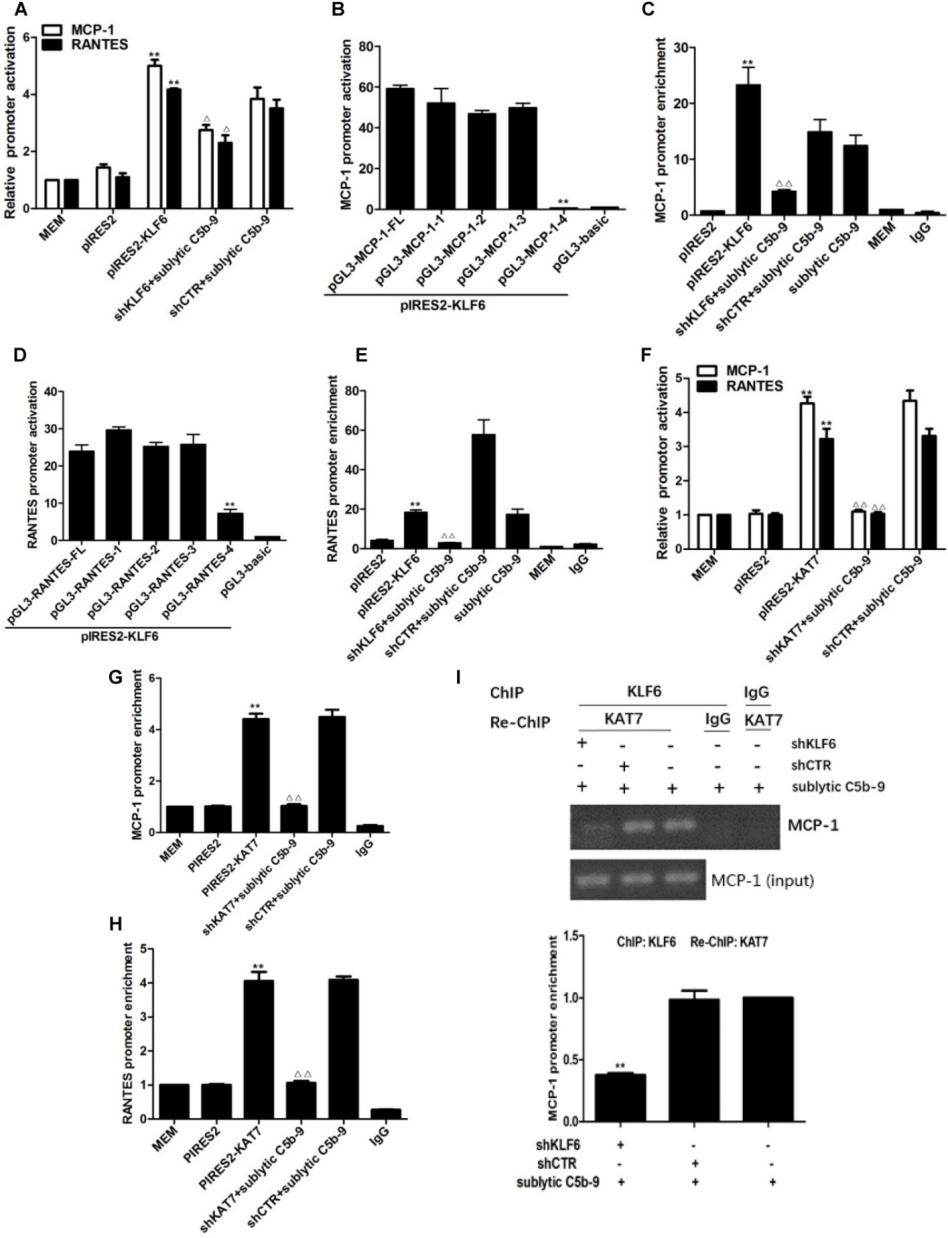

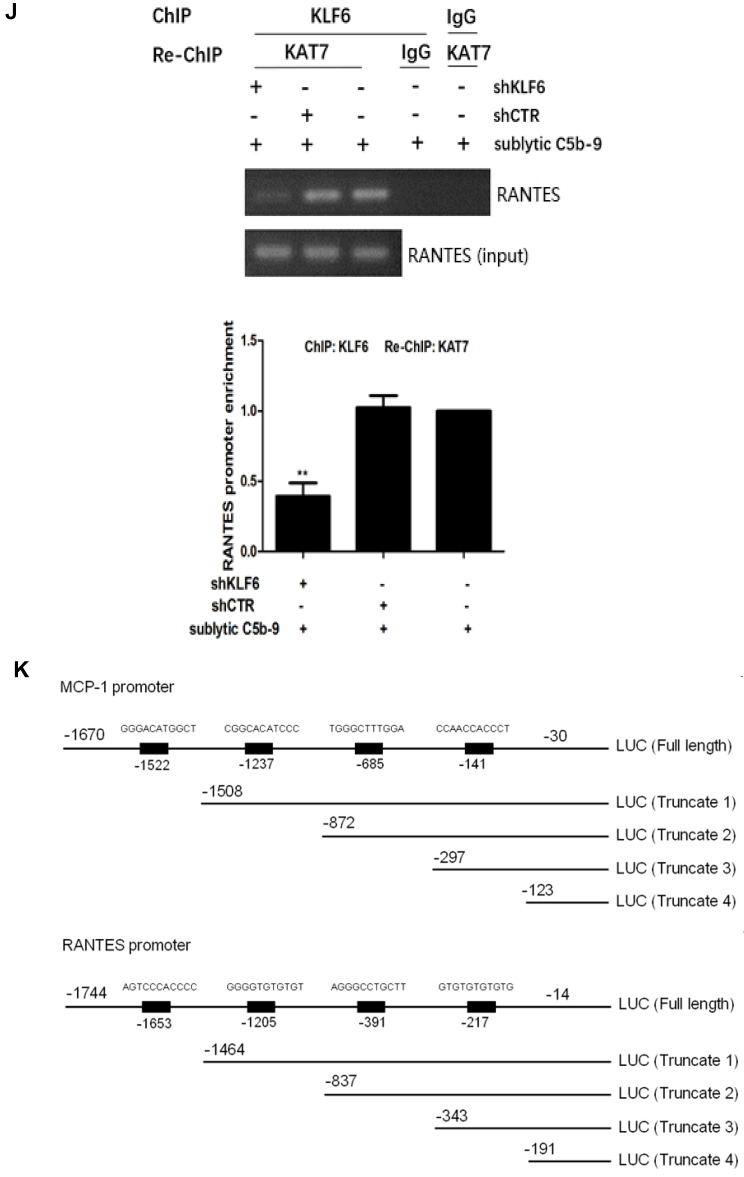
Analysis of KLF6 binding to MCP-1 and RANTES promoters together with KAT7 in GMCs attacked by sublytic C5b-9. **(A)** Luciferase activity assay of MCP-1 reporter (-1670 to -30 nt) or RANTES reporter (-1744 to -14 nt) in GMCs transfected with pIRES2, pIRES2-KLF6, shCTR or shKLF6 for 48 h and then incubated with or without sublytic C5b-9 for 5 h (**P < 0.01 vs. pIRES2; ^△^P < 0.05 vs. shCTR + sublytic C5b-9). **(B)** Luciferase activity assay of MCP-1 reporter in GMCs transfected with full length (FL) or different truncation mutants of MCP-1 reporter together with pIRES2-KLF6 for 48 h (**P < 0.01 vs. pGL3-MCP-1-FL). **(C)** ChIP analysis of KLF6 at MCP-1 promoter (-297 to -123 nt) in GMCs transfected with pIRES2, pIRES2-KLF6, shCTR or shKLF6 for 48 h and then incubated with or without sublytic C5b-9 for 5 h (**P < 0.01 vs. pIRES2; ^△△^P < 0.01 vs. shCTR + sublytic C5b-9). **(D)** Luciferase activity assay of RANTES reporter in GMCs transfected with full length (FL) or different truncation mutants of RANTES reporter together with pIRES2-KLF6 for 48 h (**P < 0.01 vs. pGL3-RANTES-FL). **(E)** ChIP analysis of KLF6 at RANTES promoter (-343 to -191 nt) in GMCs transfected with pIRES2, pIRES2-KLF6, shCTR or shKLF6 for 48 h and then incubated with or without sublytic C5b-9 for 5 h (**P < 0.01 vs. pIRES2; ^△△^P < 0.01 vs. shCTR + sublytic C5b-9). **(F)** Luciferase activity assay of MCP-1 reporter (-1670 to -30 nt) or RANTES reporter (-1744 to -14 nt) in GMCs transfected with pIRES2, pIRES2-KAT7, shCTR or shKAT7 for 48 h and then incubated with or without sublytic C5b-9 for 5 h (**P < 0.01 vs. pIRES2; ^△△^P < 0.01 vs. shCTR + sublytic C5b-9). **(G and H)** ChIP analysis of KAT7 at MCP-1 promoter (-297 to -123 nt) **(G)** or RANTES promoter (-343 to -191 nt) **(H)** in GMCs transfected with pIRES2, pIRES2-KAT7, shCTR or shKAT7 for 48 h and then incubated with or without sublytic C5b-9 for 5 h (**P < 0.01 vs. pIRES2; ^△△^P < 0.01 vs. shCTR + sublytic C5b-9). **(I and J)** ChIP analysis of KLF6 and Re-ChIP analysis of KAT7 at MCP-1 promoter (-297 to -123 nt)** (I)** or RANTES promoter (-343 to -191 nt) **(J)** in GMCs transfected with shCTR or shKLF6 for 48 h and then incubated with sublytic C5b-9 for 5 h (**P < 0.01 vs. shCTR + sublytic C5b-9). Data are representative of three independent experiments with similar results or are shown as mean ± SD from three independent experiments. **(K)** Schematic representation of rat MCP-1 and RANTES promoter deletion mutants used.

To elucidate whether sublytic C5b-9 can affect MCP-1 and RANTES gene transcription through KLF6, rat MCP-1 and RANTES proximal promoter regions were amplified by PCR, and luciferase reporter assay was performed. In comparison with other treatments, MCP-1 and RANTES promoter activity in GMCs was greatly elevated by sublytic C5b-9 stimulation (Figure S3A, S3B). Besides, KLF6 overexpression enhanced MCP-1 and RANTES promoter activity, whereas KLF6 knockdown suppressed MCP-1 and RANTES promoter activity (Figure 4A). Given that MCP-1 promoter region (-1670 to -30 nt) and RANTES promoter region (-1744 to -14 nt) contain four putative KLF6-binding elements detected in JARSPAR database (Figure 4K), we subsequently tried to identify functional elements of MCP-1 and RANTES promoters required for gene transcription in response to sublytic C5b-9-induced KLF6.

For MCP-1 promoter, sublytic C5b-9 incubation and exogenous overexpression of KLF6 elevated luciferase activity of full-length (FL) and three truncation mutant (-1508 to -30, -872 to -30 and -297 to -30 nt), but had no effect on the shortest truncation (-123 to -30 nt) mutant (Figure 4B, Figure S3C), hinting that the effective KLF6-binding element on MCP-1 promoter might locate within -297 to -123 nt and might be the putative KLF6-binding element (-141 to -131 nt). Furthermore, ChIP assay showed that the enrichment of KLF6 on MCP-1 promoter region (-297 to -123 nt) was greatly increased after KLF6 overexpression, and markedly decreased when KLF6 gene was silenced even in sublytic C5b-9 stimulation (Figure 4C). On the other hand, a similar result was also obtained in RANTES promoter, which showed that an effective KLF6-binding element was located in the region (-343 to -191 nt) of RANTES promoter and might be the putative KLF6-binding element (-217 to -207 nt) (Figure 4D, 4E, and Figure S3D). Together, these data suggest that sublytic C5b-9-induced MCP-1 and RANTES gene activation is mediated by KLF6 at transcriptional level.

Interestingly, overexpression and knockdown of KAT7 could also up-regulate and down-regulate MCP-1 and RANTES promoter activity, respectively (Figure 4F). KAT7 protein occupied the same MCP-1 promoter region (-297 to -123 nt) and RANTES promoter region (-343 to -191 nt), which contained the KLF6-binding site (Figure 4G, 4H). Therefore, to clarify whether KAT7 can bind to MCP-1 and RANTES promoters directly or via KLF6, Re-ChIP assay was performed. There was a notable reduction of KAT7 binding to MCP-1 and RANTES promoters by interfering with KLF6 gene (Figure 4I, 4J), revealing that KAT7 binds to MCP-1 and RANTES promoters indirectly in a KLF6-dependent manner. These data imply that KAT7 acts as a co-activator of KLF6 to promote transcription of MCP-1 or RANTES gene.

### KAT7 interacts and acetylates KLF6 upon sublytic C5b-9 stimulation

**Figure 5:**
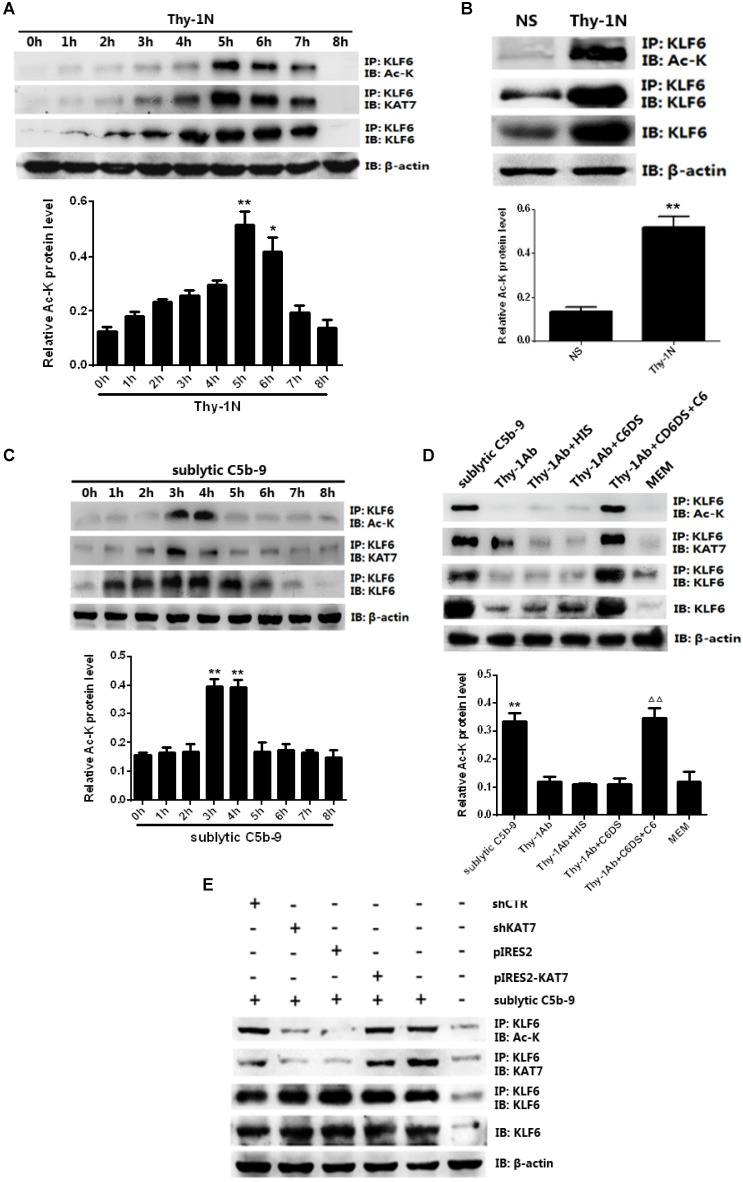
Analysis of KLF6 acetylation mediated by KAT7 in GMCs induced by sublytic C5b-9. **(A)** IB analysis of acetylated lysine (Ac-K), KLF6 and KAT7 in renal cortex of SD rats injected intravenously with Thy-1 Ab for the indicated times, assessed after IP with antibody to KLF6 (*P < 0.05, **P < 0.01 vs. 0 h). **(B)** IB analysis of acetylated lysine (Ac-K) and KLF6 in renal cortex of SD rats injected intravenously with Thy-1 Ab or normal rabbit serum (NS) for 5 h, assessed after IP with antibody to KLF6 (**P < 0.01 vs. NS). **(C)** IB analysis of acetylated lysine (Ac-K), KLF6 and KAT7 in GMCs stimulated with sublytic C5b-9 for the indicated times, assessed after IP with antibody to KLF6 (**P < 0.01 vs. 0 h). **(D)** IB analysis of acetylated lysine (Ac-K), KLF6 and KAT7 in GMCs stimulated with sublytic C5b-9, Thy-1 Ab, Thy-1 Ab + HIS, Thy-1 Ab + C6DS, Thy-1 Ab + C6DS + C6, or MEM for 3 h, assessed after IP with antibody to KLF6 (**P < 0.01 vs. Thy-1 Ab, Thy-1 Ab + HIS, Thy-1 Ab + C6DS, and MEM; ^△△^P < 0.01 vs. Thy-1 Ab + C6DS). **(E)** IB analysis of acetylated lysine (Ac-K), KLF6 and KAT7 in GMCs transfected with pIRES2, pIRES2-KAT7, shCTR or shKAT7 for 48 h and then incubated with sublytic C5b-9 for 3 h, assessed after IP with antibody to KLF6. Data are representative of three independent experiments with similar results or are shown as mean ± SD from three independent experiments.

KAT7 is a specific co-factor with intrinsic acetyltransferase activity (36, and the activity of KLF6 can be affected by acetylation 37, but whether KAT7 can acetylate KLF6 remains unknown. IP assay exhibited an interaction between KAT7 and KLF6 as well as an up-regulation of KLF6 acetylation level both *in vivo and in vitro* (Figure 5A-D). More specifically, the levels of KAT7-KLF6 binding and KLF6 acetylation were increased in a time-dependent manner, peaking at 5 h in the kidney of Thy-1N rats (Figure 5A, 5B). Besides, sublytic C5b-9 induced KAT7-KLF6 binding and KLF6 acetylation in a time-dependent manner with a peak level at 3h in cultured GMCs (Figure 5C, 5D). Additionally, knockdown of KAT7 by specific shRNA not only obviously inhibited the interaction between KAT7 and KLF6, but also markedly reduced KLF6 acetylation in GMCs stimulated by sublytic C5b-9 (Figure 5E). Taken together, we pinpointed for the first time that KLF6 is able to be acetylated by KAT7 in GMCs exposed to sublytic C5b-9.

### KLF6 acetylation at residue K100 by KAT7 promotes MCP-1 and RANTES gene transcription

**Figure 6:**
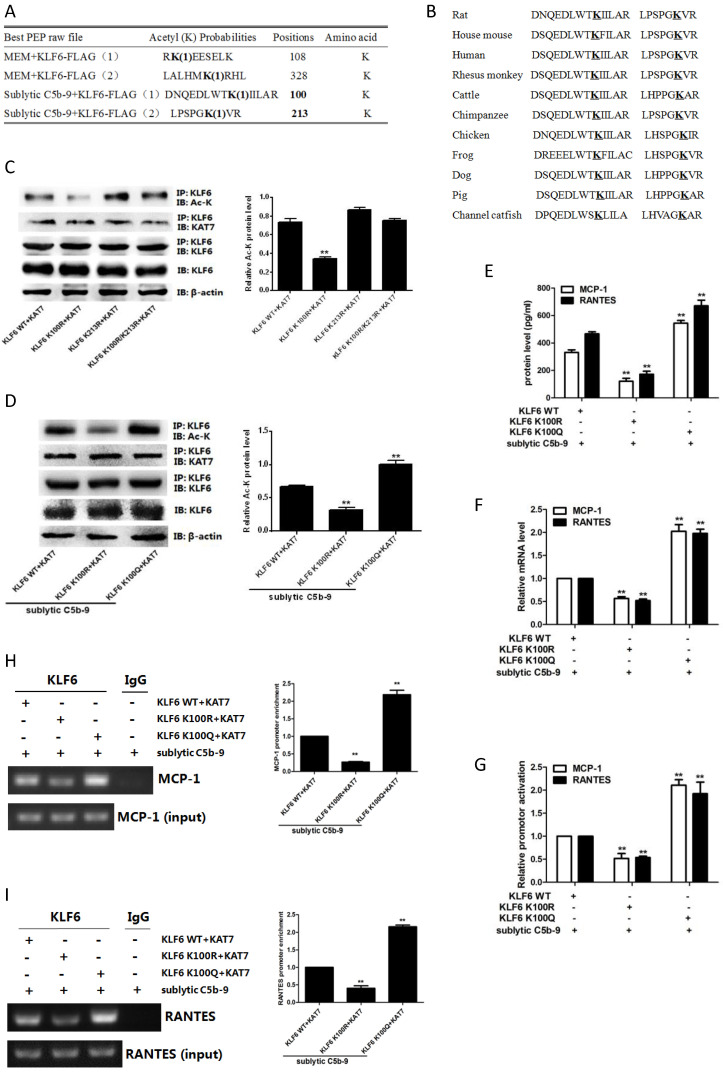
Effect of KLF6 acetylation on MCP-1 and RANTES gene transcription in GMCs exposed to sublytic C5b-9.** (A)** Mass spectrometry analysis of acetylated lysine residues in KLF6 protein upon sublytic C5b-9 stimulation.** (B)** Sequence alignment of potential acetylation sites in KLF6. The conserved lysine residues are in bold and underlined. **(C)** IB analysis of acetylated lysine (Ac-K), KLF6 and KAT7 in GMCs transfected with vector encoding WT or mutant KLF6 (K100R, K213R and K100R/K213R) along with vector encoding KAT7 for 48 h, assessed after IP with antibody to KLF6. **(D)** IB analysis of acetylated lysine (Ac-K), KLF6 and KAT7 in GMCs transfected with vector encoding WT or mutant KLF6 (K100R and K100Q) along with vector encoding KAT7 for 48 h and then incubated with sublytic C5b-9 for 3 h, assessed after IP with antibody to KLF6.** (E)** ELISA of MCP-1 and RANTES in supernatants of GMCs transfected with vector encoding WT or mutant KLF6 (K100R and K100Q) along with vector encoding KAT7 for 48 h and then incubated with sublytic C5b-9 for 5 h. **(F)** qPCR analysis of MCP-1 and RANTES mRNA in GMCs transfected with vector encoding WT or mutant KLF6 (K100R and K100Q) along with vector encoding KAT7 for 48 h and then incubated with sublytic C5b-9 for 5 h. **(G)** Luciferase activity assay of MCP-1 reporter (-1670 to -30 nt) or RANTES reporter (-1744 to -14 nt) in GMCs transfected with vector encoding WT or mutant KLF6 (K100R and K100Q) along with vector encoding KAT7 for 48 h and then incubated with sublytic C5b-9 for 5 h. **(H and I)** ChIP analysis of KLF6 at MCP-1 promoter (-297 to -123 nt) **(H)** or RANTES promoter (-343 to -191 nt) **(I)** in GMCs transfected with vector encoding WT or mutant KLF6 (K100R and K100Q) along with vector encoding KAT7 for 48 h and then incubated with sublytic C5b-9 for 5 h. **P < 0.01 vs. KLF6 WT + KAT7 + sublytic C5b-9. Data are representative of three independent experiments with similar results or are shown as mean ± SD from three independent experiments.

Given that KAT7 interacted with and acetylated KLF6, we wanted to identify the acetylation sites of KLF6. To this purpose, mass spectrometry analysis was used and we found that two lysines (K100 and K213) of rat KLF6 were acetylated in GMCs attacked by sublytic C5b-9 (Figure 6A). The two candidate lysine residues and their surrounding regions are highly evolutionarily conserved among other species (Figure 6B). To further pinpoint which lysine site is acetylated by KAT7, we mutated the two sites separately or together with change of lysine (K) to arginine (R), which are mimics of acetylation-deficient KLF6, and then co-transfected different KLF6 mutants with KAT7 expression vector into GMCs. The data displayed that only K100R mutant remarkably reduced the level of KLF6 acetylation (Figure 6C). Next, we generated K100R mutant by changing lysine (K) to glutamine (Q), which is a mimic of hyper-acetylated KLF6. Consistent with the above result, K100R notably decreased KLF6 acetylation, whereas K100Q significantly increased KLF6 acetylation (Figure 6C). Collectively, these findings demonstrate that K100 is the major acetylation site of KLF6 by KAT7 and is conserved during the evolution.

To further explore the function of KLF6 acetylation, we investigated the effect of KLF6-K100 acetylation on MCP-1 and RANTES gene expression. As shown in Figure 6E, 6F, both protein and mRNA levels of MCP-1 and RANTES were greatly repressed by the expression of acetylation-deficient mutant KLF6-K100R, and the expression of MCP-1 and RANTES was up-regulated by the presence of hyper-acetylated mutant KLF6-K100Q. Besides, KLF6-K100R mutant showed an attenuated luciferase activity of MCP-1 and RANTES promoters, whereas KLF6-K100Q mutant exhibited an enhanced MCP-1 and RANTES promoter activity (Figure 6G). Meanwhile, MCP-1 and RANTES promoters were less occupied by KLF6 when using KLF6-K100R mutant, but each promoter was more occupied by KLF6 when using KLF6-K100Q mutant (Figure 6H, 6I). Overall, these findings strongly confirm that KAT7-mediated acetylation at K100 of KLF6 boosts the transcription of MCP-1 and RANTES genes.

### Knockdown of renal KAT7 or KLF6 gene ameliorates the production of MCP-1 and RANTES and pathological change in Thy-1N rats

Given that KAT7 and KLF6 were able to promote MCP-1 and RANTES synthesis in sublytic C5b-9-induced GMCs as shown above, it is tempting to know whether KAT7 and KLF6 play a role in renal MCP-1 and RANTES production and GMCs proliferation of Thy-1N rats. Thy-1N model rats pretreated with LV-shKAT7 or LV-shKLF6 via renal artery perfusion Figure S4A, S4B) had decreased KAT7 or KLF6 expression at 4 h in renal tissue Figure S4C).

**Figure 7:**
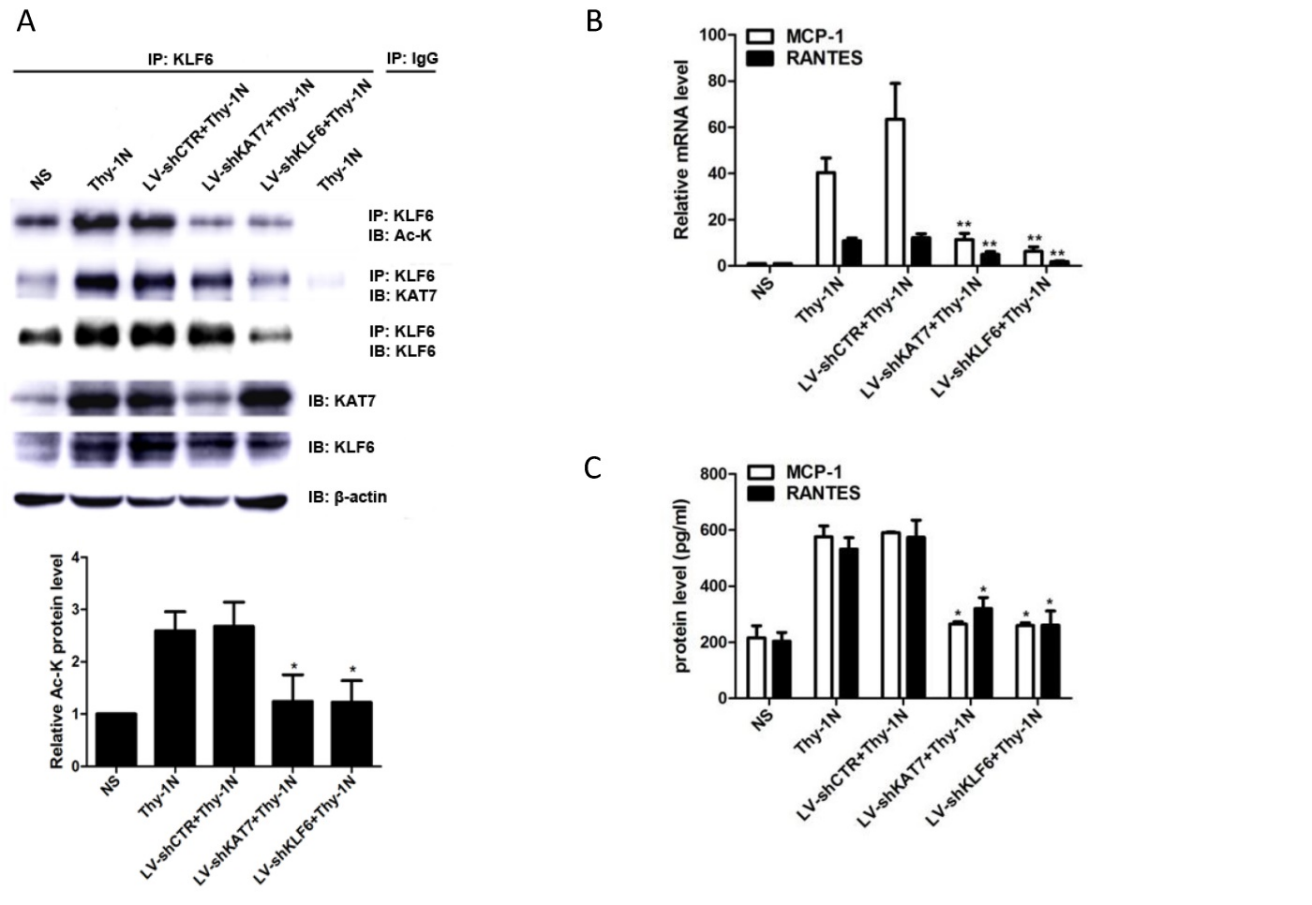

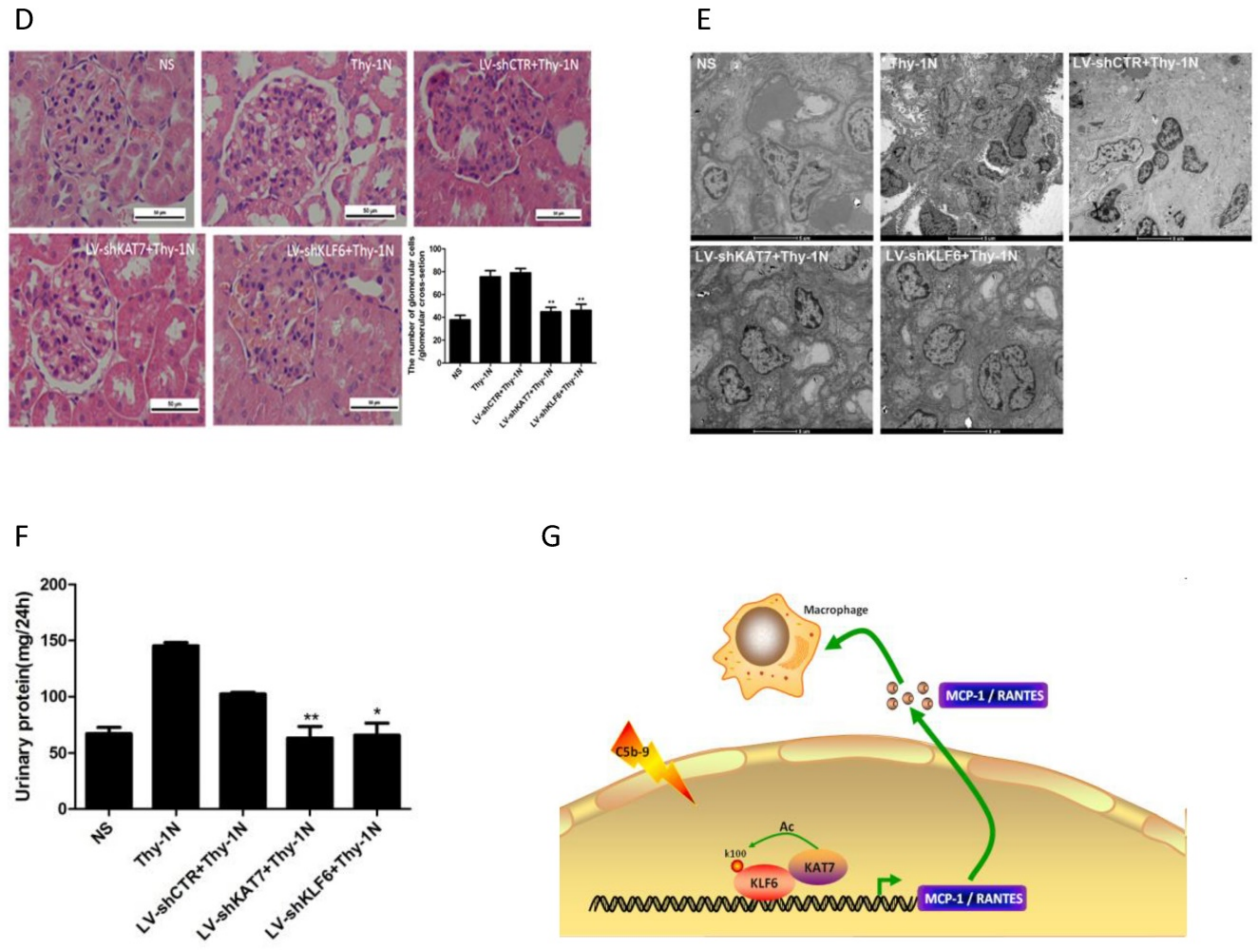
Effect of renal KAT7 and KLF6 gene knockdown on MCP-1 and RANTES production and pathological change in Thy-1N rats. LV-shRNA was infused into SD rat kidney via renal artery perfusion, and 96 h later these rats were injected intravenously with Thy-1 Ab for 4 h **(A-C)** or 7 days** (D-F)**.** (A)** IB analysis of acetylated lysine (Ac-K), KLF6 and KAT7 in rat renal cortex, assessed after IP with IgG or antibody to KLF6. **(B)** qPCR analysis of MCP-1 and RANTES mRNA in rat renal cortex.** (C)** ELISA of MCP-1 and RANTES in rat renal cortex supernatants.** (D)** H&E staining of glomerular cells in rat renal cortex paraffin sections (×400). **(E)** Electron microscopy to diagnostic ultrastructural pathology in rat renal cortex (×5000). **(F)** Urine protein analysis of total contents of urinary protein excretion (milligrams per 24 h). **(G)** A putative scheme for the molecular mechanism of pro-inflammatory chemokines production in rat GMCs induced by sublytic C5b-9. In response to sublytic C5b-9, KLF6, KAT7, MCP-1, and RANTES are all up-regulated. The elevated KAT7 can interact with and acetylate KLF6 at lysine 100 to strengthen the combination of KLF6 to MCP-1 and RANTES promoters, resulting in further augment MCP-1 and RANTES gene transcription and expression. *P < 0.05, **P < 0.01 vs. LV-shCTR + Thy-1N. Data are representative of three independent experiments with similar results or are shown as mean ± SD from three independent experiments.

The pretreatment with LV-shKAT7 obviously inhibited the level of KLF6 acetylation at 4 h (Figure 7A). The pretreatments with LV-shKAT7 or LV-shKLF6 significantly reduced MCP-1 and RANTES expression at 4 h (Figure 7B, 7C). Besides, LV-shKAT7 or LV-shKLF6 also greatly diminished the number of total glomerular cells (Figure 7D), GMCs proliferation and ECM accumulation (Figure 7E) on day 7 after Thy-1N model was established (Here, GMCs proliferation is defined as more than three mesangial cells in the mesangium. ECM accumulation represents more mesangial matrix produced by mesangial cells). Furthermore, LV-shKAT7 or LV-shKLF6 treatment could also markedly lessen the content of urinary protein on day 7 (Figure 7F). The findings *in vivo* disclose that KAT7 and KLF6 promote the synthesis of MCP-1 or RANTES, and aggravate renal lesions of Thy-1N rats.

## Discussion

Glomerulonephritis is characterized by glomerular inflammation and is a major cause of end-stage renal disease. Although the deposition of complement membrane attack complex (C5b-9) has been observed in the glomeruli of patients with MsPGN e.g. IgA nephropathy (38, 39, the role of C5b-9 in the pathogenesis of MsPGN has not been fully elucidated until now. Rat Thy-1N is a wide-spreadly used animal model that recapitulates the main features of human MsPGN 4. The initial mechanism of this nephritis is the binding of injecting anti-Thy-1 antibody to rat Thy-1 antigen on GMCs membrane, triggering complement system activation, inflammatory response development, and subsequent GMCs injury 30, 34, 40. Our previous experiments have revealed that sublytic C5b-9 complex is the major mediator of renal inflammation and GMCs damage in the rats with Thy-1N 8, 31. Sublytic C5b-9 induces the release of pro-inflammatory cytokines (IL-6, IL-23, and IL-36a) by GMCs 7, 8 and GMCs apoptosis 31 or proliferation 30. In this study, we found that the expression of chemokines MCP-1 and RANTES increased obviously in renal tissue of Thy-1N rats (*in vivo*) and in GMCs stimulated with sublytic C5b-9 (*in vitro*), indicating that the up-regulation of MCP-1 and RANTES may contribute to the recruitment of macrophages into glomeruli in the early stage of Thy-1N. However, the mechanism by which sublytic C5b-9 induces MCP-1 and RANTES synthesis in GMCs of Thy-1N rats is unknown.

We explored downstream agents of sublytic C5b-9 and upstream regulators of MCP-1 and RANTES. It has been reported that sublytic C5b-9 assembly in nucleated cells membrane interacts with Gi protein, inducing Ras activation. Ras then activates Raf-1, which in turn leads to MEK1 and ERK1 activation, causing activating several transcription factors during the inflammation 41, 42. Besides, previous experiments have proved that the regulation of MCP-1 and RANTES expression is related to specific transcription factors. For instance, AP-1 and NF-κB have a key role in controlling MCP-1 gene transcription 9. NF-κB, AP-1, C/EBP, IRF3, Sp1, and Fli-1 play a pivotal role in promoting RANTES expression 15, 43. In the present study, we found that transcription factor KLF6 was induced by sublytic C5b-9 stimulation and transcriptionally activated MCP-1 and RANTES. On one hand, the up-regulation or down-regulation of KLF6 expression correspondingly caused the rise or reduction of MCP-1 and RANTES expression in GMCs incubated with sublytic C5b-9. On the other hand, knockdown of renal KLF6 not only led to decrease MCP-1 and RANTES expression, but also resulted in less GMCs proliferation, ECM accumulation, and urinary protein level in Thy-1N rats. More importantly, our luciferase and ChIP data confirmed that KLF6 transactivated MCP-1 and RANTES by binding to MCP-1 promoter region (-297 to -123 nt) and RANTES promoter region (-343 to -191 nt). To our knowledge, this is first study to identify KLF6 as a direct transcriptional activator of the inflammatory chemokines MCP-1 and RANTES under sublytic C5b-9 conditions.

KLF6 mediates various cellular processes, including proliferation, differentiation, and apoptosis 44. Dysregulated KLF6 expression contributes to the pathologies of numerous diseases such as obesity, hepatic fibrosis, cancer, and inflammatory responses 17, 45. Besides, previous study has demonstrated that overexpression of KLF6 in cultured proximal tubule cells exposed to high glucose reduces the expression of epithelial markers while increasing mesenchymal markers 19. Our previous study suggests that KLF6 expression is induced in cultured GMCs in the setting of sublytic C5b-9-mediated apoptosis 16. Combined with our present study highlighted that KLF6 played a role in the production of pro-inflammatory chemokines in Thy-1N, we have reasons for believing that KLF6 might be very important in the progression of Thy-1N.

Similar to our results, KLF6 behaves as a mild activator as demonstrated for several target genes including p21^CIP1/WAF1^, TGFβ1, and its receptors type I and II, among others 37, 45. Although KLF6 is a moderate activator, its acidic domain is able to mediate high level of transcription when fused to a heterologous DNA-binding domain, suggesting that under particular situations e.g. posttranslational modifications, KLF6 mediates stronger transcriptional activation 18. The kinase S6K1 has been involved in KLF6 phosphorylation, which is necessary for induction of TGFβ1 gene transcription 46. KLF6 is ubiquitinated in cultured cells following exposure to DNA damaging agents 47. Moreover, KLF6 binds CBP and P/CAF and is subsequently acetylated at K209, resulting in enhanced transcriptional activity 37. Based on reports mentioned above, we sought to determine whether other acetyltransferases would regulate the transaction of KLF6 in GMCs attacked by sublytic C5b-9.

We screened the expression of twelve histone acetyltransferase (HAT) members belong to the Gcn5-related N-acetyltransferase (GNAT) superfamily and the MYST family, and found that KAT7 not only up-regulated both in renal tissue of Thy-1N rats and in cultured GMCs exposed to sublytic C5b-9, but also expressed a little earlier than KLF6, MCP-1, and RANTES. Moreover, interfering with renal KAT7 ameliorated MCP-1 and RANTES production, GMCs proliferation, ECM accumulation, and urinary protein secretion in Thy-1N rats. Mechanistically, we found that although KAT7 advanced promoter activity and mRNA level of MCP-1 and RANTES, KAT7 did not bind to the gene promoters directly. Instead, KAT7 was recruited to the promoter regions by interaction with transcription activator KLF6, implying that KAT7 is a co-activator of KLF6. On the other hand, knockdown of KAT7 reduced KLF6 acetylation both in kidney of Thy-1N rats and in sublytic C5b-9-induced GMCs. More importantly, KLF6 was acetylated by KAT7 at lysine residue 100 (K100) through mass spectrometry. Intriguingly, acetylation-deficient mutant KLF6-K100R decreased the KLF6 binding ability and transcriptional activity, leading to lessen MCP-1 and RANTES production, whereas hyper-acetylated mutant KLF6-K100Q increased the KLF6 binding ability and transcriptional activity, leading to raise MCP-1 and RANTES production. Therefore, this study identified KAT7 as a previously undiscovered acetyltransferase of transcription factor KLF6, thereby initiating its activation effect. Because an acetyltransferase affects gene expression in a wide spectrum, it is of interest to identify other potential transcription factors targets of KAT7 in MCP-1 and RANTES regulation. Apart from transcription factors, histone H3 and H4 are acetylated by KAT7 25-26. Another question remains unresolved is if the histone acetylation by KAT7 contributes to MCP-1 and RANTES gene transcription. It is reported that KAT7 promotes histone H4 acetylation at IL-6 and TGF-β promoter to activate their transcriptions 29. Therefore, we speculate that KAT7 could promote histone acetylation of MCP-1 and RNATES promoter, which might alter the accession of KLF6 to the chromatin and elevate KLF6 transcriptional activity. Given that the documents on KAT7 are very limited, addressing these puzzles would provide a more sophisticated understanding on the role of KAT7 in sublytic C5b-9-induced GMCs.

In summary, our studies revealed that KLF6, KAT7, MCP-1, and RANTES are increased both in renal tissue of Thy-1N rats and in sublytic C5b-9-induced GMCs. Mechanically, sublytic C5b-9-elevated KLF6 recruited KAT7 by protein-protein interaction to bind MCP-1 promoter region (-297 to -123 nt) and RANTES promoter region (-343 to -191 nt) in a KLF6-dependent manner, in turn activating MCP-1 and RANTES gene transcription. In this process, KLF6 was acetylated by KAT7 at lysine residue 100, thereby contributing to up-regulation of MCP-1 and RANTES production in sublytic C5b-9-induced GMCs. Furthermore, silencing of renal KAT7 or PCAF not only suppressed MCP-1 and RANTES synthesis, but also inhibited GMCs proliferation, ECM accumulation, and proteinuria production in Thy-1N rats (Figure 7G). Taken together, our findings indicate that sublytic C5b-9-induced MCP-1 and RANTES production is due to KAT7-mediated KLF6 acetylation in GMCs of Thy-1N rats, which might provide a new insight into the pathogenesis of rat Thy-1N and human MsPGN.

## Supplementary Material

Supplementary figures and tables.

## Abbreviations

Thy-1N: Thy-1 nephritis
MsPGN: mesangioproliferative glomerulonephritis
GMCs: glomerular mesangial cells
ECM: extracellular matrix
KLF6: Kruppel-like factor 6
KAT7: lysine acetyltransferase 7
HAT: histone acetyltransferase
HIS: heat-inactivated serum
C6DS: complement C6-deficient serum
NS: normal rabbit serum

## References

[1] Jiang M, Xiao Z, Rong L (2016). Twenty-eight-year review of childhood renal diseases from renal biopsy data: A single centre in China. Nephrology (Carlton).

[2] Jin M, Yin Z, Wei K (2019). Metanephric mesenchyme-derived Foxd1+ mesangial precursor cells alleviate mesangial proliferative glomerulonephritis. J Mol Med (Berl).

[3] Onda K, Ohsawa I, Ohi H (2011). Excretion of complement proteins and its activation marker C5b-9 in IgA nephropathy in relation to renal function. BMC Nephrol.

[4] Bai J, Wu L, Chen X (2018). Suppressor of Cytokine Signaling-1/STAT1 Regulates Renal Inflammation in Mesangial Proliferative Glomerulonephritis Models. Front Immunol.

[5] Huang K, Li R, Wei W (2018). Sirt1 activation prevents anti-Thy 1.1 mesangial proliferative glomerulonephritis in the rat through the Nrf2/ARE pathway. Eur J Pharmacol.

[6] Brandt J, Pippin J, Schulze M (1996). Role of the complement membrane attack complex (C5b-9) in mediating experimental mesangioproliferative glomerulonephritis. Kidney Int.

[7] Zhang J, Xie M, Xia L (2018). Sublytic C5b-9 Induces IL-23 and IL-36a Production by Glomerular Mesangial Cells via PCAF-Mediated KLF4 Acetylation in Rat Thy-1 Nephritis. J Immunol.

[8] Zhang J, Li Y, Shan K (2014). Sublytic C5b-9 induces IL-6 and TGF-β1 production by glomerular mesangial cells in rat Thy-1 nephritis through p300-mediated C/EBPβ acetylation. FASEB J.

[9] Bianconi V, Sahebkar A, Atkin SL, Pirro M (2018). The regulation and importance of monocyte chemoattractant protein-1. Curr Opin Hematol.

[10] Mohs A, Kuttkat N, Reißing J (2017). Functional role of CCL5/RANTES for HCC progression during chronic liver disease. J Hepatol.

[11] Haller H, Bertram A, Nadrowitz F, Menne J (2016). Monocyte chemoattractant protein-1 and the kidney. Curr Opin Nephrol Hypertens.

[12] Krensky AM, Ahn YT (2007). Mechanisms of disease: regulation of RANTES (CCL5) in renal disease. Nat Clin Pract Nephrol.

[13] Liu H, Zhang XP, Yi ZW (2013). Efficacy of antisense monocyte chemoattractant protein-1 (MCP-1) in a rat model of mesangial proliferative glomerulonephritis. Ren Fail.

[14] Panzer U, Schneider A, Wilken J (1999). The chemokine receptor antagonist AOP-RANTES reduces monocyte infiltration in experimental glomerulonephritis. Kidney Int.

[15] Lennard Richard ML, Sato S, Suzuki E (2014). The Fli-1 transcription factor regulates the expression of CCL5/RANTES. J Immunol.

[16] Rane MJ, Zhao Y, Cai L (2019). Krϋppel-like factors (KLFs) in renal physiology and disease. EBioMedicine.

[17] Kim GD, Das R, Goduni L (2016). Kruppel-like Factor 6 Promotes Macrophage-mediated Inflammation by Suppressing B Cell Leukemia/Lymphoma 6 Expression. J Biol Chem.

[18] Andreoli V, Gehrau RC, Bocco JL (2010). Biology of Krüppel-like factor 6 transcriptional regulator in cell life and death. IUBMB Life.

[19] Holian J, Qi W, Kelly DJ (2008). Role of Kruppel-like factor 6 in transforming growth factor-beta1-induced epithelial-mesenchymal transition of proximal tubule cells. Am J Physiol Renal Physiol.

[20] Qi W, Holian J, Tan CY (2011). The roles of Kruppel-like factor 6 and peroxisome proliferator-activated receptor-γ in the regulation of macrophage inflammatory protein-3α at early onset of diabetes. Int J Biochem Cell Biol.

[21] Xu K, Zhou Y, Qiu W (2011). Activating transcription factor 3 (ATF3) promotes sublytic C5b-9-induced glomerular mesangial cells apoptosis through up-regulation of Gadd45α and KLF6 gene expression. Immunobiology.

[22] Miyamoto S, Suzuki T, Muto S (2003). Positive and negative regulation of the cardiovascular transcription factor KLF5 by p300 and the oncogenic regulator SET through interaction and acetylation on the DNA-binding domain. Mol Cell Biol.

[23] Zhang W, Bieker JJ (1998). Acetylation and modulation of erythroid Krüppel-like factor (EKLF) activity by interaction with histone acetyltransferases. Proc Natl Acad Sci U S A.

[24] Yan MS, Turgeon PJ, Man HJ (2018). Histone acetyltransferase 7 (KAT7)-dependent intragenic histone acetylation regulates endothelial cell gene regulation. J Biol Chem.

[25] Quintela M, Sieglaff DH, Gazze AS (2019). HBO1 directs histone H4 specific acetylation, potentiating mechano-transduction pathways and membrane elasticity in ovarian cancer cells. Nanomedicine.

[26] Feng Y, Vlassis A, Roques C (2016). BRPF3-HBO1 regulates replication origin activation and histone H3K14 acetylation. EMBO J.

[27] Iizuka M, Susa T, Takahashi Y (2013). Histone acetyltransferase Hbo1 destabilizes estrogen receptor α by ubiquitination and modulates proliferation of breast cancers. Cancer Sci.

[28] Long C, Lai Y, Li J (2018). LPS promotes HBO1 stability via USP25 to modulate inflammatory gene transcription in THP-1 cells. Biochim Biophys Acta Gene Regul Mech.

[29] Gao S, Qi X, Li J, Sang L (2017). Upregulated KAT7 in synovial fibroblasts promotes Th17 cell differentiation and infiltration in rheumatoid arthritis. Biochem Biophys Res Commun.

[30] Yu T, Wang L, Zhao C (2019). Sublytic C5b-9 Induces Proliferation of Glomerular Mesangial Cells via ERK5/MZF1/RGC-32 Axis Activated by FBXO28-TRAF6 Complex. J Cell Mol Med.

[31] He F, Zhou M, Yu T (2016). Sublytic C5b-9 triggers glomerular mesangial cell apoptosis in rat Thy-1 nephritis via Gadd45 activation mediated by Egr-1 and p300-dependent ATF3 acetylation. J Mol Cell Biol.

[32] Pippin JW, Durvasula R, Petermann A (2003). DNA damage is a novel response to sublytic complement C5b-9-induced injury in podocytes. J Clin Invest.

[33] Shankland SJ, Pippin JW, Couser WG (1999). Complement (C5b-9) induces glomerular epithelial cell DNA synthesis but not proliferation in vitro. Kidney Int.

[34] Jiang X, Zhang J, Xia M (2010). Role of activating transcription factor 3 (ATF3) in sublytic C5b-9-induced glomerular mesangial cell apoptosis. Cell Mol Immunol.

[35] Date D, Das R, Narla G (2014). Kruppel-like Transcription Factor 6 Regulates Inflammatory Macrophage Polarization. J Biol Chem.

[36] Iizuka M, Susa T, Tamamori-Adachi M (2017). Intrinsic ubiquitin E3 ligase activity of histone acetyltransferase Hbo1 for estrogen receptor α. Proc Jpn Acad Ser B Phys Biol Sci.

[37] Li D, Yea S, Dolios G (2005). Regulation of Kruppel-like factor 6 tumor suppressor activity by acetylation. Cancer Res.

[38] Stangou M, Alexopoulos E, Pantzaki A (2008). C5b-9 glomerular deposition and tubular alpha3beta1-integrin expression are implicated in the development of chronic lesions and predict renal function outcome in immunoglobulin A nephropathy. Scand J Urol Nephrol.

[39] Turnberg D, Cook HT (2005). Complement and glomerulonephritis: new insights. Curr Opin Nephrol Hypertens.

[40] Lu Y, Mei Y, Chen L (2019). The role of transcriptional factor D-site-binding protein in circadian CCL2 gene expression in anti-Thy1 nephritis. Cell Mol Immunol.

[41] Fosbrink M, Niculescu F, Rus H (2005). The role of c5b-9 terminal complement complex in activation of the cell cycle and transcription. Immunol Res.

[42] Lu N, Malemud CJ (2019). Extracellular Signal-Regulated Kinase: A Regulator of Cell Growth, Inflammation, Chondrocyte and Bone Cell Receptor-Mediated Gene Expression. Int J Mol Sci.

[43] Pocock J, Gómez-Guerrero C, Harendza S (2003). Differential activation of NF-kappa B, AP-1, and C/EBP in endotoxin-tolerant rats: mechanisms for in vivo regulation of glomerular RANTES/CCL5 expression. J Immunol.

[44] McConnell BB, Yang VW (2010). Mammalian Krüppel-like factors in health and diseases. Physiol Rev.

[45] Kim Y, Ratziu V, Choi SG (1998). Transcriptional activation of transforming growth factor beta1 and its receptors by the Kruppel-like factor Zf9/core promoter-binding protein and Sp1. Potential mechanisms for autocrine fibrogenesis in response to injury. J Biol Chem.

[46] Lee SJ, Yang EK, Kim SG (2006). Peroxisome proliferator-activated receptor-gamma and retinoic acid X receptor alpha represses the TGFbeta1 gene via PTEN-mediated p70 ribosomal S6 kinase-1 inhibition: role for Zf9 dephosphorylation. Mol Pharmacol.

[47] Banck MS, Beaven SW, Narla G (2006). KLF6 degradation after apoptotic DNA damage. FEBS Lett.

