# Supplementary material for: KLF6 Acetylation Promotes Sublytic C5b-9-Induced Production of MCP-1 and RANTES in Experimental Mesangial Proliferative Glomerulonephritis
Source: Int J Biol Sci. 2020 Jun 20;16(13):2340–56. doi: 10.7150/ijbs.46573 (PMC7378648; doi:10.7150/ijbs.46573)
Supplement: Supplementary file 1 — Supplementary figures and tables. [file ijbsv16p2340s1.pdf]

Table S1: Specific primers used in plasmids construction

Name	Primer	Sequence, 5'→3'
pIRES2-KLF6	BglII top	GAAGATCTAAATATTGCGTGGGCTCGG
	EcoRI bottom	CCGGAATTCTGGGTGCTATGCCGCTTCT
pIRES2-KAT7	PstI top	AACTGCAGATGGCGATAGGTGTAAAG
	SacII bottom	TCCCCGCGGCAGAACAGTGCTGAGGG
pIRES2-KLF6-FLAG	XhoI top	CCGCTCGAGATGGATTACAAGGATGACGACGATAAGAAACT TTCACCTGCG
	EcoRI bottom	CCGGAATTCAAGAGGCATCTCTGA
MCP-1 promoter	-1670 MluI top	CGACGCGTGAGCCAAACTCACAACGA
	-1508 MluI top	CGACGCGTGGAACCAGAGTGTCCTAGA
	-872 MluI top	CGACGCGTTTGAACGTAACATGGTGAT
	-297 MluI top	CGACGCGTCTTCATTTGCTCCCAGTA
	-123 MluI top	CGACGCGTTCCACCCTCTGGCTTACA
	-30 XhoI bottom	CCCTCGAGGTGAGAGTTGGCTGGTTT
RANTES promoter	-1744 MluI top	CGACGCGTGTCGGTAGATGAACGG
	-1464 MluI top	CGACGCGTCAGAGATGGGATGATTGT
	-837 MluI top	CGACGCGTATTCCCACAAAGACTCA
	-343 MluI top	CGACGCGTTCTACCCCCCATTACTAT
	-191 MluI top	CGACGCGTGTGTGTGTTTTCATTTTC
	-14 XhoI bottom	CCCTCGAGGAGACTGTGGAAGATGC

Primer sequences corresponding to rat genes were designed and underlined.

Table S2: Specific primers used in PCR analysis

Name	Primer	Sequence, 5'→3'
ATF2 (RT-PCR)	FW	ACGGCAGTGGATTGG
	RV	TGGCACGGAAAGGTC
HAT1 (RT-PCR)	FW	TATTGCTGGTAGCCTGTC
	RV	TCCACCGCACTCTTAT
KAT2A (RT-PCR)	FW	ATCGGTGGGATTTGCTT
	RV	CCTGCTTGGTGTCCGTGT
KAT2B (RT-PCR)	FW	GGAAAGCCTATGGTTGA
	RV	TGGGAAATGCGTGAG
KAT5 (RT-PCR)	FW	ACCTTGCCAATCCCG
	RV	CATCTTCGTTGTCCTGGTT
KAT6A (RT-PCR)	FW	TCAGCCCAGAGCAAGG
	RV	TGCCAAGCCCTCAAAT
KAT6B (RT-PCR)	FW	CATTGCCTTACAGCCACTC
	RV	GGGAACGCCCATAGATT
KAT7 (RT-PCR)	FW	GCACTGAGGAACCCGCCTAT
	RV	ACCGCCTGTTCCGTTTCAGA
KAT8 (RT-PCR)	FW	AACAGGCGACTGGACG
	RV	CACGGTGACTTCTGGTTCG
KAT13A (RT-PCR)	FW	ATCCGACCCTGCGAACC
	RV	TATGTGAGTCTGGGTTCG
KAT13B (RT-PCR)	FW	GAGACAGATACGCCAAATAA
	RV	CCTGAAAGGTCGTGCC
KAT13D (RT-PCR)	FW	AACTCCTTCTGCCTCC
	RV	CCAGGGTTTGATTGC
GAPDH (RT-PCR)	FW	GCACTGAGGAACCCGCCTAT
	RV	ACCGCCTGTTCCGTTTCAGA
MCP-1 (qPCR)	FW	CTTCTGGGCCTGTTGTTCAC
	RV	GGCATTAACTGCATCTGGCT
RANTES (qPCR)	FW	GCCCACGTGAAGGAGTATTT
	RV	CCACTTCTTCTCTGGGTTGG
KLF6 (qPCR)	FW	GCTCCCACTTGAAAGCACAT
	RV	GCTTTCGGAAGTGTCTGGTC
KAT7 (qPCR)	FW	GCACTGAGGAACCCGCCTAT
	RV	ACCGCCTGTTCCGTTTCAGA
β-actin (qPCR)	FW	TCACCCACACTGTGCCCATCTATGA
	RV	CATCGGAACCGCTCATTGCCGATAG
MCP-1 (ChIP)	FW	GCAGATTCAAACTTCCAC
	RV	TGAGAGTTGGCTGGTTT
RANTES (ChIP)	FW	CTGAGGATGAAGGGAAGGA
	RV	CTGGCTGCTGTCAGAAAAT

FW: forward; RV: reverse.

Specific primers were designed to amplify corresponding genes in different experiments.

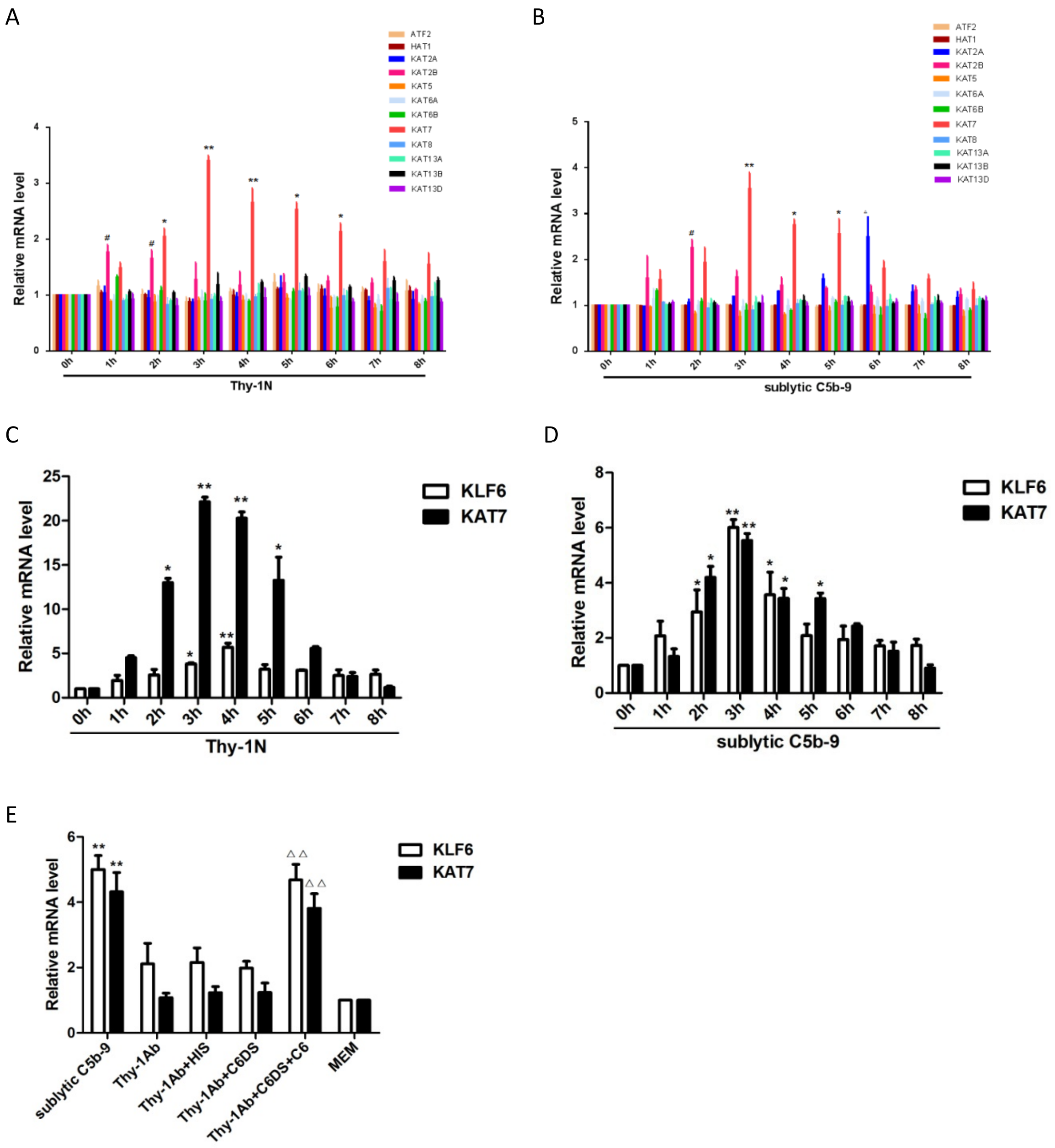

Figure S1. Expression of twelve HATs members and KLF6 mRNA both in renal tissue of Thy-1N rats and in GMCs upon sublytic C5b-9 stimulation. **(A)** RT-PCR analysis of twelve HAT members mRNA in renal cortex of SD rats injected intravenously with Thy-1 Ab for the indicated times ($^{\#}P < 0.05$, $^{*}P < 0.05$, $^{**}P < 0.01$ vs. 0 h). **(B)** RT-PCR analysis of twelve HAT members mRNA in GMCs stimulated with sublytic C5b-9 for the indicated times ($^{\#}P < 0.05$, $^{*}P < 0.05$, $^{**}P < 0.01$ vs. 0 h). **(C)** qPCR analysis of KLF6 and KAT7 mRNA in renal cortex of SD rats injected intravenously with Thy-1 Ab for the indicated times ($^{*}P < 0.05$, $^{**}P < 0.01$ vs. 0 h). **(D)** qPCR analysis of KLF6 and KAT7 mRNA in GMCs stimulated with sublytic C5b-9 for the indicated times ($^{*}P < 0.05$, $^{**}P < 0.01$ vs. 0 h). **(E)** qPCR analysis of KLF6 and KAT7 mRNA in GMCs stimulated with sublytic C5b-9, Thy-1 Ab, Thy-1 Ab + HIS, Thy-1 Ab + C6DS, Thy-1 Ab + C6DS + C6, or MEM for 3 h ($^{**}P < 0.01$ vs. Thy-1 Ab, Thy-1 Ab + HIS, Thy-1 Ab + C6DS, and MEM; $^{\triangle\triangle}P < 0.01$ vs. Thy-1 Ab + C6DS). Data from three independent experiments are presented as mean ± SD.

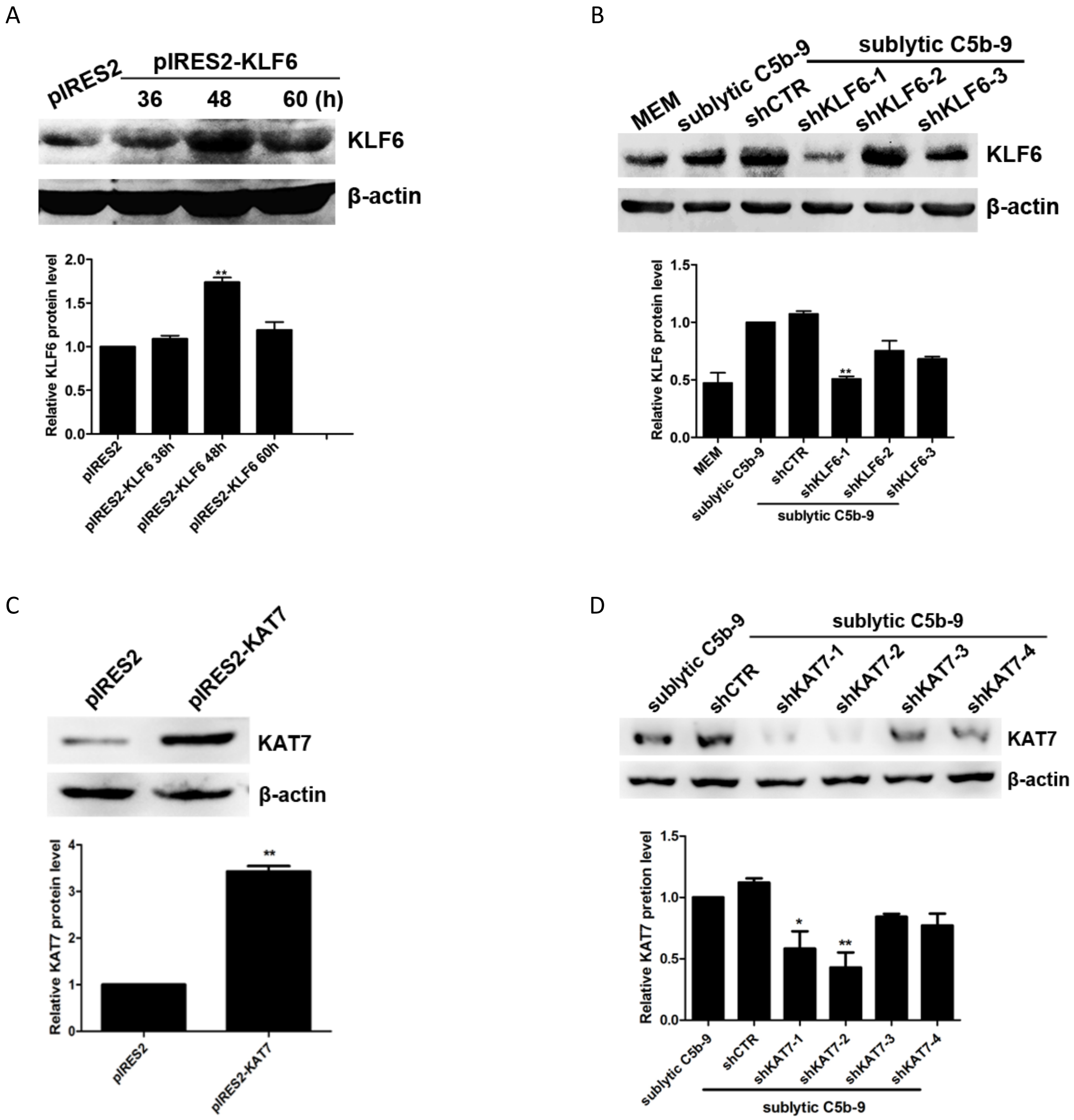

Figure S2. Expression of KLF6 and KAT7 in GMCs transfected with corresponding plasmids. **(A)** IB analysis of KLF6 and β-actin in GMCs transfected with control vector (pIRES2) or vector encoding KLF6 (pIRES2-KLF6) for 36, 48, or 60 h (**P < 0.01 vs. pIRES2). **(B)** IB analysis of KLF6 and β-actin in GMCs transfected with control shRNA (shCTR), or three classes of shRNA targeting KLF6 (shKLF6) for 48 h and then incubated with sublytic C5b-9 for 3 h (**P < 0.01 vs. shCTR + sublytic C5b-9). **(C)** IB analysis of KAT7 and β-actin in GMCs transfected with control vector (pIRES2) or vector encoding KAT7 (pIRES2-KAT7) for 48 h (**P < 0.01 vs. pIRES2). **(D)** IB analysis of KAT7 and β-actin in GMCs transfected with control shRNA (shCTR), or four classes of shRNA targeting KAT7 (shKAT7) for 48 h and then incubated with sublytic C5b-9 for 3 h (**P < 0.01 vs. shCTR + sublytic C5b-9). Data are representative of three independent experiments with similar results or are shown as mean ± SD from three independent experiments.

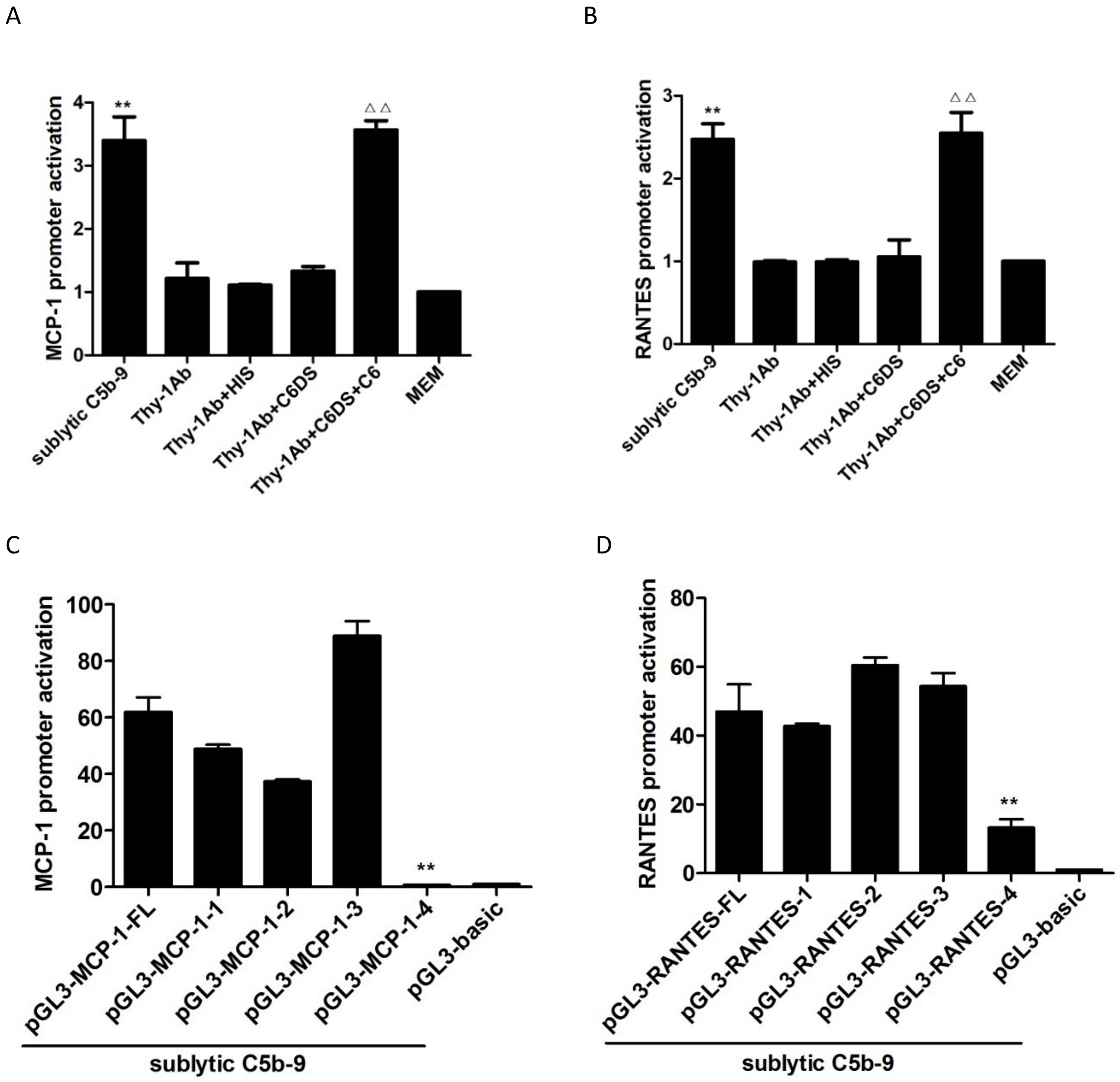

Figure S3. Effect of sublytic C5b-9 on MCP-1 and RANTES promoter activity. **(A)** Luciferase activity assay of MCP-1 reporter (-1670 to -30 nt) in GMCs stimulated with sublytic C5b-9, Thy-1 Ab, Thy-1 Ab + HIS, Thy-1 Ab + C6DS, Thy-1 Ab + C6DS + C6, or MEM for 5 h (**P < 0.01 vs. Thy-1 Ab, Thy-1 Ab + HIS, Thy-1 Ab + C6DS, and MEM; △△P < 0.01 vs. Thy-1 Ab + C6DS). **(B)** Luciferase activity assay of RANTES reporter (-1744 to -14 nt) in GMCs stimulated with sublytic C5b-9, Thy-1 Ab, Thy-1 Ab + HIS, Thy-1 Ab + C6DS, Thy-1 Ab + C6DS + C6, or MEM for 5 h (**P < 0.01 vs. Thy-1 Ab, Thy-1 Ab + HIS, Thy-1 Ab + C6DS, and MEM; △△P < 0.01 vs. Thy-1 Ab + C6DS). **(C)** Luciferase activity assay of MCP-1 reporter in GMCs transfected with full length (FL) or different truncation mutants of MCP-1 reporter for 48 h and then incubated with sublytic C5b-9 for 5 h (**P < 0.01 vs. pGL3-MCP-1-FL). **(D)** Luciferase activity assay of RANTES reporter in GMCs transfected with full length (FL) or different truncation mutants of RANTES reporter for 48 h and then incubated with sublytic C5b-9 for 5 h (**P < 0.01 vs. pGL3-RANTES-FL). Data from three independent experiments are presented as mean ± SD.

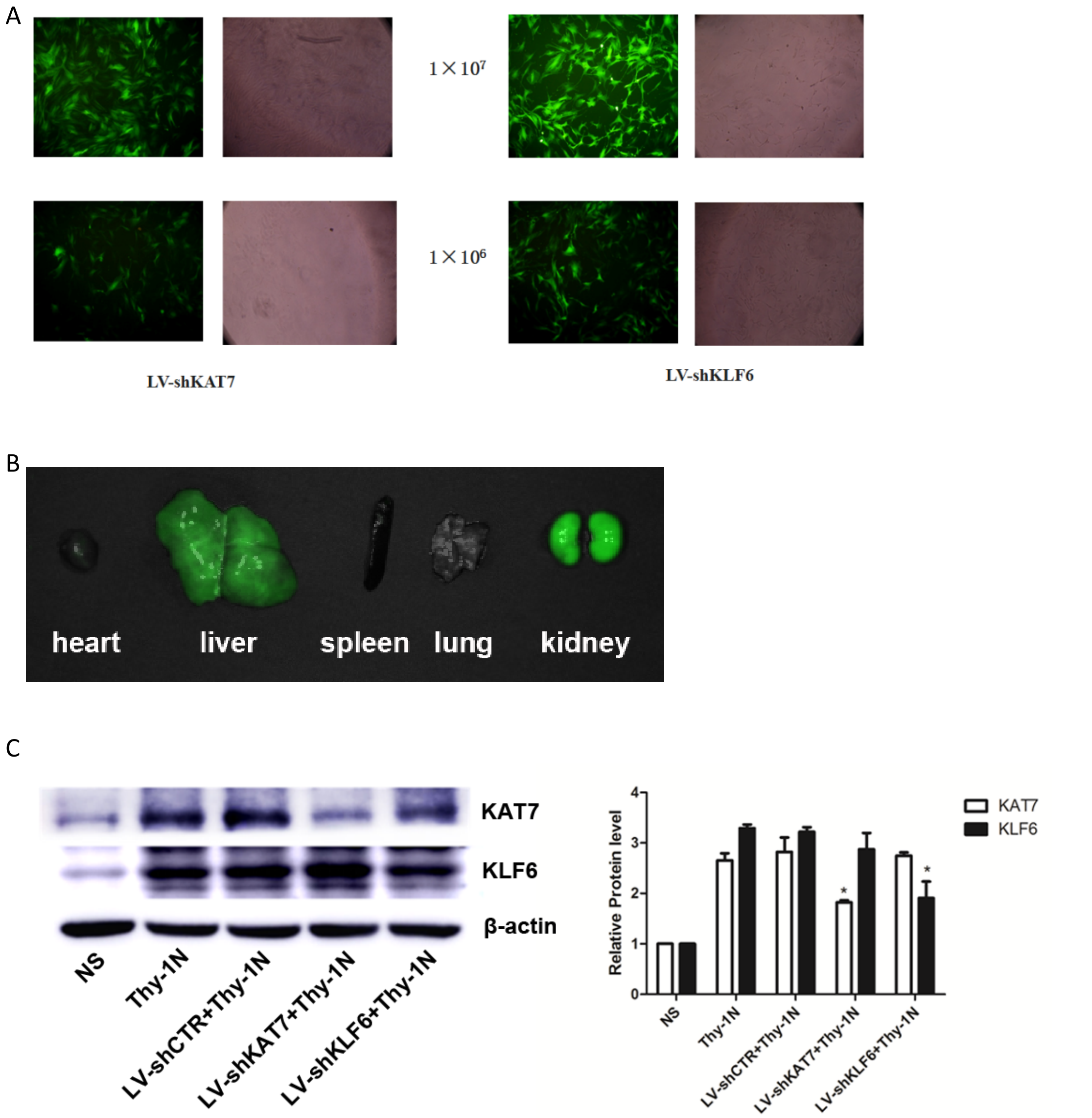

Figure S4. Effect of LV-shKAT7 and LV-shKLF6 on the expression of corresponding genes in renal tissue of Thy-1N rats. **(A)** GMCs were cultured with lentivirus (LV) at the titer of 1×10^6TU/ml or 1×10^7TU/ml. **(B)** On delivery of the LV-shCTR through renal artery, EGFP expression in different organs was observed by Caliper IVIS in vivo imaging system. **(C)** LV-shRNA was infused into SD rat kidney via renal artery perfusion, and 96 h later these rats were injected intravenously with Thy-1 Ab for 4 h. IB analysis of KAT7, KLF6 and β-actin in rat renal cortex (*$P < 0.05$ vs. LV-shCTR + Thy-1N). Data are representative of three independent experiments with similar results or are shown as mean ± SD from three independent experiments.
